# Metabolic engineering of *Rhodotorula toruloides* IFO0880 improves C16 and C18 fatty alcohol production from synthetic media

**DOI:** 10.1186/s12934-022-01750-3

**Published:** 2022-02-19

**Authors:** J. Carl Schultz, Shekhar Mishra, Emily Gaither, Andrea Mejia, Hoang Dinh, Costas Maranas, Huimin Zhao

**Affiliations:** 1 Department of Chemical and Biomolecular Engineering, Carl R. Woese Institute for Genomic Biology, University of Illinois at Urbana-Champaign, Urbana, IL 61801 USA; 2 U.S. Department of Energy Center for Bioenergy and Bioproducts Innovation (CABBI), Urbana, IL 61801 USA; 3 Department of Chemical Engineering, Pennsylvania State University, University Park, PA 16802 USA; 4 Departments of Chemistry, Biochemistry, and Bioengineering, University of Illinois at Urbana-Champaign, Urbana, IL 61801 USA

**Keywords:** *Rhodotorula toruloides*, Fatty alcohols, Metabolic engineering, Lipidomics, CRISPR/Cas9

## Abstract

**Background:**

The oleaginous, carotenogenic yeast *Rhodotorula toruloides* has been increasingly explored as a platform organism for the production of terpenoids and fatty acid derivatives. Fatty alcohols, a fatty acid derivative widely used in the production of detergents and surfactants, can be produced microbially with the expression of a heterologous fatty acyl-CoA reductase. Due to its high lipid production, *R. toruloides* has high potential for fatty alcohol production, and in this study several metabolic engineering approaches were investigated to improve the titer of this product.

**Results:**

Fatty acyl-CoA reductase from *Marinobacter aqueolei* was co-expressed with SpCas9 in *R. toruloides* IFO0880 and a panel of gene overexpressions and Cas9-mediated gene deletions were explored to increase the fatty alcohol production. Two overexpression targets (*ACL1* and *ACC1*, improving cytosolic acetyl-CoA and malonyl-CoA production, respectively) and two deletion targets (the acyltransferases *DGA1* and *LRO1*) resulted in significant (1.8 to 4.4-fold) increases to the fatty alcohol titer in culture tubes. Combinatorial exploration of these modifications in bioreactor fermentation culminated in a 3.7 g/L fatty alcohol titer in the *LRO1*Δ mutant. As *LRO1* deletion was not found to be beneficial for fatty alcohol production in other yeasts, a lipidomic comparison of the *DGA1* and *LRO1* knockout mutants was performed, finding that *DGA1* is the primary acyltransferase responsible for triacylglyceride production in *R. toruloides*, while *LRO1* disruption simultaneously improved fatty alcohol production, increased diacylglyceride and triacylglyceride production, and increased glucose consumption.

**Conclusions:**

The fatty alcohol titer of fatty acyl-CoA reductase-expressing *R. toruloides* was significantly improved through the deletion of *LRO1*, or the deletion of *DGA1* combined with overexpression of *ACC1* and *ACL1*. Disruption of *LRO1* surprisingly increased both lipid and fatty alcohol production, creating a possible avenue for future study of the lipid metabolism of this yeast.

**Supplementary Information:**

The online version contains supplementary material available at 10.1186/s12934-022-01750-3.

## Background

Long-chain alcohols are widely used as constituents of cosmetics, surfactants, and detergents [1]. These compounds have traditionally been produced chemically, through hydrolysis and hydrogenation of plant or animal fats, or directly synthesized by oligomerization of ethylene followed by oxidation of the resulting olefin [2]. In the United States, synthesis from ethylene is the predominant method [2]; however, this process requires the use of non-renewable petroleum feedstocks as well as the highly energy-intensive production of ethylene by steam cracking at 750–950 °C [3]. The use of microbes as cellular factories for chemical conversion has been increasingly investigated for many chemical products including fuels [4], pharmaceuticals [5], and various other value-added bioproducts [6] from renewable feedstocks at approximately ambient temperature and pressure, creating the potential for significant energy savings and reduced carbon emissions compared to traditional chemical synthesis approaches.

Fatty alcohols are natively produced in many organisms as a component of natural waxes by reduction of fatty acids or fatty acyl-CoA by carboxylic acid reductases (CARs) or fatty acyl-CoA reductases (FARs), respectively [1]. These fatty alcohol-producing genes have been expressed in a variety of heterologous microbial hosts including *Escherichia coli* [7], *Synechocystis* species [8], *Saccharomyces cerevisiae*, *Yarrowia lipolytica* [9], and *Rhodotorula toruloides* [10], enabling fatty alcohol production by microbial fermentation. Strikingly, fatty alcohol titers and yields remain far below those obtained for related compounds such as lipids and fatty acids [11, 12]. Significant metabolic rewiring of genetically tractable organisms such as *E. coli* and *S. cerevisiae* has led to noteworthy increases in the fatty alcohol titers obtained, with *E. coli* producing 6.3 g/L with the deletion of thioesterase gene *tesCB*, lactate dehydrogenase gene *ldhA*, acetate kinase gene *ackA*, and phosphate acetyltransferase gene *pta* [13]*. S. cerevisiae* produced 6.0 g/L of fatty alcohols using FAR from *Mus musculus* after 9 distinct genome edits were combined including targets for deletion and overexpression, and replacing the negative regulator of the *GAL1* promoter used for FAR expression with a positive regulator [14]. Oleaginous yeasts such as *Y. lipolytica* and *R. toruloides* are promising candidate hosts for fatty alcohol bioproduction owing to their high levels of acyl-CoA production, which is natively used to produce lipids. However, metabolic engineering efforts in these less domesticated yeasts are less sophisticated, with *Y. lipolytica* producing 5.8 g/L of fatty alcohols using FAR from *Marinobacter aquaeolei* (MaFAR) after overexpression of *DGA1*, mutagenesis of the *Mga2* regulator, and expression of 2 copies of FAR [9]. Although production of 8 g/L fatty alcohols has been reported from growth of *R. toruloides* on YP-sucrose media in a fed-batch fermentation also using MaFAR [10], no metabolic engineering efforts towards production of fatty alcohols have been reported to date, likely due to the lack of advanced gene editing tools compared to *E. coli* and *S. cerevisiae*. Nevertheless, the impressive results achieved so far in oleaginous yeasts demonstrate their potential as production hosts for fatty acid derivatives. With additional metabolic engineering work, the fatty alcohol titers produced in these yeast species can doubtlessly be further increased.

Recently, the genetic engineering toolkit for *R. toruloides* has been improved significantly with development of CRISPR gene editing [15–17] and RNA interference [18], and characterization of many promoters [19–21]. In this study, we applied the CRISPR/Cas9 gene editing system we previously developed in combination with overexpression of native and heterologous genes to create and explore a broad selection of fatty alcohol-producing upregulation and knockout mutants in *R. toruloides* IFO0880. We observed that improving the supply of precursor metabolites, blocking the formation of TAGs, and disrupting genes potentially involved in fatty alcohol degradation all improved the fatty alcohol titer of the producing strain. Beneficial modifications were then explored combinatorially at the culture tube and bioreactor scale, and lipidomic analysis of engineered strains shed light on the differing roles of the acyltransferases *DGA1* and *LRO1* in the lipid metabolism of *R. toruloides*.

## Results

### Selection of promoter and FAR gene for fatty alcohol production

Various FAR genes have been tested for production of fatty alcohols in different yeast species. D’Espaux and coworkers compared the previously used *Marinobacter aqueaolei* VT8, *Tyto alba*, and *Mus musculus* FARs in *S. cerevisiae*, finding that MmFAR1 yielded the highest titer [14], while in *Yarrowia lipolytica*, Cordova and coworkers compared FAR genes from *Apis mellifera*, *Homo sapiens*, *Arabidopsis thaliana*, and *M. aqueaolei*, finding that FAR from *M. aqueaolei* (maqu_2220 or MaFAR) showed the best performance [9]. In *R. toruloides*, FARs from *M. aqueaolei*, *T. alba*, *A. thaliana*, *A. mellifera*, *G. gallus*, and *A. domesticus* were recently compared, with MaFAR also giving the highest titer in this species [22]. Owing to its high activity in both *R. toruloides* and *Y. lipolytica*, MaFAR was selected for use in this study. It is possible this enzyme functions particularly well in oleaginous yeasts as its preferred substrates are the highly plentiful C16 and C18 acyl-CoA [23].

**Fig. 1:**
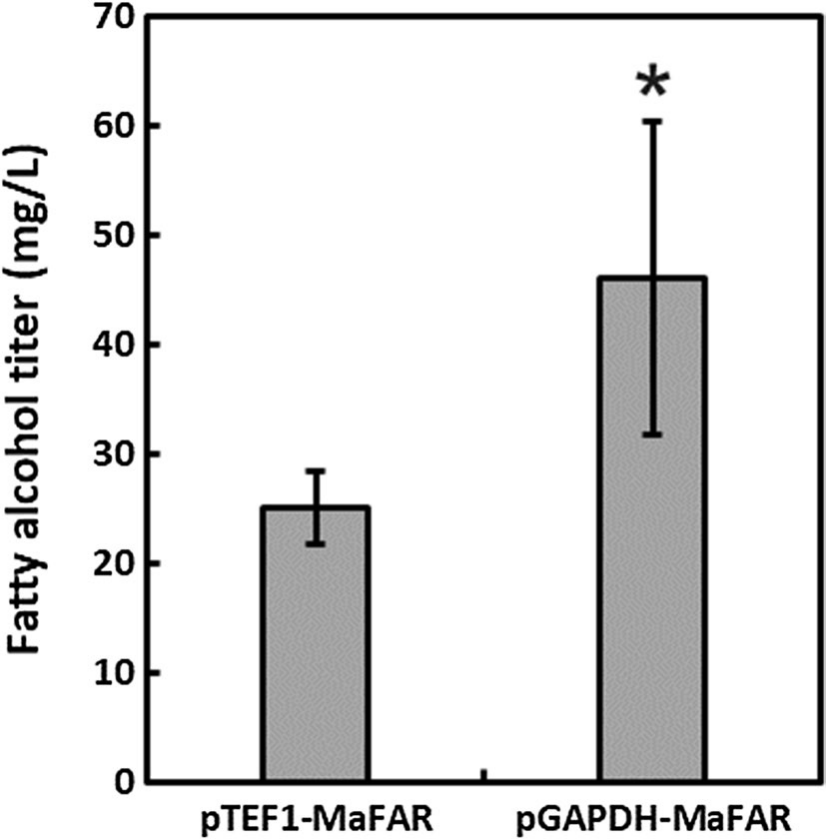
Fatty alcohol titers resulting from pTEF1- and pGAPDH-driven MaFAR expression in *R. toruloides* IFO0880. Error bars represent ± standard deviation of biological duplicates. Presence of an asterisk indicates statistically significant difference in titer (*p* < 0.05, student *t*-test)

The *GAPDH*, or *TDH3*, promoter is known to be a strong promoter in the model yeast *S. cerevisiae* and the native version has frequently been used for heterologous gene expression in *R. toruloides* [10, 24, 25]*.* While a recent study of constitutive promoters in *R. toruloides* identified various promoters stronger than *pGAPDH* such as the transcription elongation factor promoter (*pTEF1*) and the adenine nucleotide transporter promoter (*pANT1*) in a GFP expression assay, when *pANT1* and *pGAPDH* were compared for fatty alcohol production in another recent study in *R. toruloides*, *pGAPDH*-driven expression resulted in a four-fold greater titer of 50 mg/L [19, 22]. 1-hexadecanol and 1-octadecenol constituted approximately 25% each of the total fatty alcohols, while the remaining 50% were 1-octadecanol. We compared *pTEF1*- and *pGAPDH*-driven *MaFAR* expression and similarly found *pGAPDH* to give a higher titer of fatty alcohols (Fig. 1). Therefore, *pGAPDH* was used to drive *MaFAR* expression in all subsequent experiments.

### Optimization of CRISPR/Cas9 gene editing in *R. toruloides* IFO0880

The creation of knockout strains has long been an essential step for the metabolic engineering of microbial organisms, typically with the goal of preventing carbon flux from traveling through undesirable pathways, increasing the flux available for the intended product [1]. Homologous recombination (HR)-based gene disruption was previously used in *R. toruloides* to delete *PEX10* with the goal of improving lipid production [12]. Due to the very low efficiency of HR in the wild-type *R. toruloides*, the nonhomologous end-joining (NHEJ)-associated gene *KU70* was first disrupted. Subsequently an antibiotic marker with 1 kb homology arms targeting the *PEX10* locus was transformed and *PEX10* was disrupted with 27% efficiency.

CRISPR/Cas9-mediated gene editing is advantageous compared to HR-based strategies in that only a 20 bp guide RNA (gRNA) must be cloned rather than 1 kb homology arms, and this editing can be performed in a wild-type *R. toruloides* strain rather than a *KU70*-deficient strain, which is known to suffer from much lower transformation efficiency [12]. We previously reported CRISPR/Cas9 gene editing in the *R. toruloides* strain NP11 with > 95% efficiency in the deletion of the carotenogenic reporter gene *CAR2* [15], with pPGK1-driven SpCas9 expression and gRNA expression driven by a fusion 5S rRNA-tRNA^Arg^ promoter. We attempted to directly transfer this system into *R. toruloides* IFO0880, which has become the better characterized strain due to various tool development, omics and modeling studies [19, 26–28], but found there was no gene editing activity. The *PGK1* promoter elements of the strains NP11 and IFO0880 are 92% similar and the two strains overall share ~ 95% genetic similarity [25]. However, our results suggest the strains are dissimilar enough that promoter elements are not always interchangeable between them.

CRISPR/Cas9-mediated gene editing was also previously reported in the *R. toruloides* strain IFO0880, using genetic elements from this strain, with *pGAPDH*-driven SpCas9 expression and tRNA^Tyr^-driven gRNA expression, albeit with a lower efficiency of 46% in the deletion of *CAR2* [16]. Reasoning that the problem with our design was related to the use of promoter elements from NP11, we next attempted to improve the efficiency of our system by testing a variety of medium (*pGAPDH*, *p27*) and strong (*pTEF1*, *pANT1*, and *p17*, driving expression of an unknown gene [19]) promoters driving SpCas9 expression and either tRNA^Tyr^ or 5S rRNA-tRNA^Tyr^-driven gRNA expression in the knockout of *CAR2*. We first randomly integrated the Cas9 expression cassette to IFO0880, then selected two colonies from each Cas9 transformation to receive the gRNA expression cassette subsequently. In contrast to FAR expression, Cas9 gene editing activity benefitted substantially from replacing *pGAPDH* with stronger promoters such as *pTEF1*, *pANT1*, and *p17,* while the medium strength *p27* resulted in similar efficiency as *pGAPDH* (Table 1). In most cases both gRNA expression systems performed similarly. The combination of *pANT1* and 5S-tRNA^Tyr^ showed 100% editing efficiency in both colonies screened and was chosen to drive Cas9 and gRNA expression, respectively, for subsequent metabolic engineering efforts. The sequences for the codon-optimized SpCas9, MaFAR, and all other synthetic genes used in this study are shown in Additional file 1: Table S1.

**Table 1:** *CAR2* disruption efficiency obtained from combinatorial optimization of Cas9 and gRNA promoters for high-efficiency gene disruption, screening 2 random Cas9 integrants per Cas9 promoter tested

	tRNA^Tyr^ (%)	5S-tRNA^Tyr^
pGAPDH-SpCas9	2	n.d
	13	
p27-SpCas9	13	17%
	9	10%
pTEF1-SpCas9	93	97%
	96	85%
pANT1-SpCas9	0	100%
	2	100%
p17-SpCas9	100	100%
	4	9%

### Metabolic engineering to improve fatty alcohol production in *R. toruloides*

**Fig. 2:**
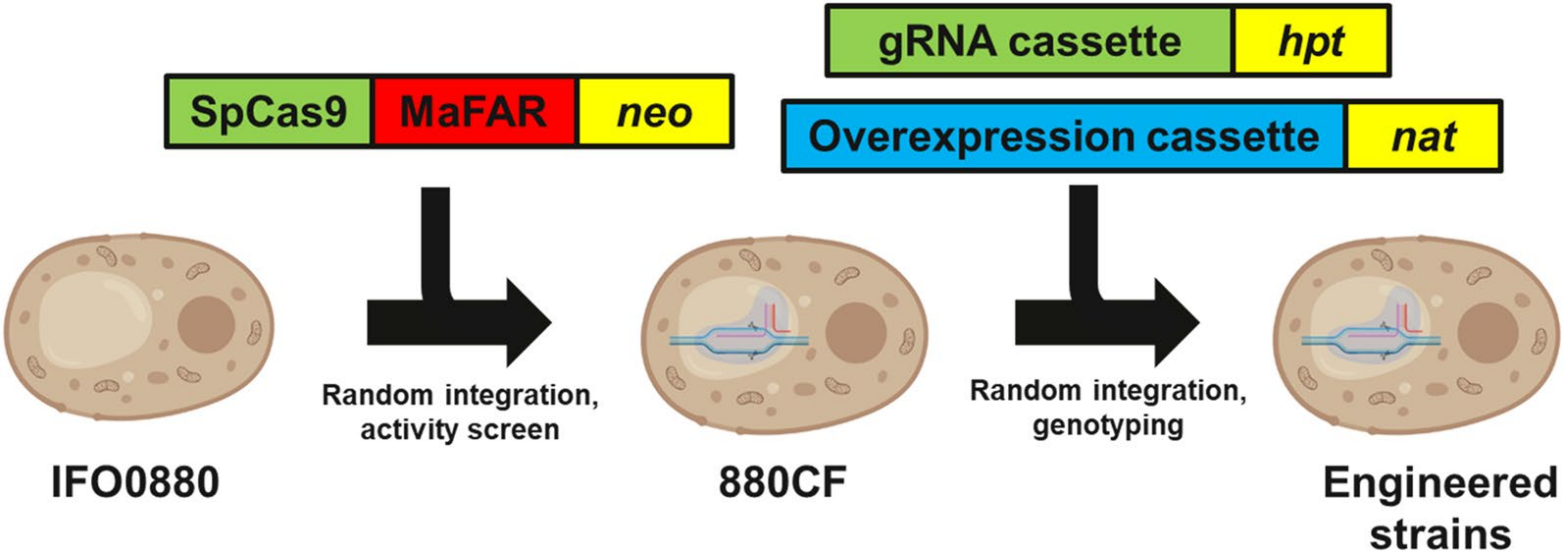
Schematic overview of the strain engineering process employed in this study. SpCas9 and MaFAR were co-integrated to create a platform strain, 880CF, followed by integration of gRNA cassettes to create knockout mutants and gene overexpression cassettes for overexpression strains. *neo* gene encoding aminoglycoside 3'-phosphotransferase (G418 resistance), *hpt* gene encoding hygromycin phosphotransferase (hygromycin resistance), *nat* gene encoding nourseothricin acetyltransferase (nourseothricin resistance)

To create a platform strain for our engineering efforts, a combined *pGAPDH-MaFAR* and *pANT1-SpCas9* expression cassette was randomly integrated to the *R. toruloides* IFO0880 genome. Random integration mutants were screened for FAR activity, and Cas9 activity in the deletion of *CAR2* as reported previously [14, 15], and a clone (subsequently referred to as 880CF) was isolated with 99% + efficiency for *CAR2* deletion upon transformation of a *CAR2*-targeting gRNA and producing a fatty alcohol titer of 50 mg/L in glass culture tubes with a 10% dodecane overlay. Subsequent engineering was performed by randomly integrating either a gRNA expression cassette targeting the beginning of an ORF for NHEJ-mediated gene disruption, or a *p17*-driven overexpression cassette to 880CF. G418 was used as the selection marker for the combined Cas9 and MaFAR integration, while hygromycin and nourseothricin were used for gRNA and overexpression cassette integrations, respectively. A schematic overview of the strain engineering process used in this study is provided in Fig. 2. To mitigate the issue of variable expression resulting from random integration, three colonies were screened for each overexpression target, the fatty alcohol titers were measured and the clone with the highest titer (corresponding to the most optimal expression level for beneficial overexpression targets) was preserved and tested in replicate for the final comparison. For deletions, genomic DNA at the target locus (a predicted high-efficiency cut site in the first 5–10% of the gene ORF) was sequenced and clones containing a frame-shift mutation were considered to have the target gene disrupted. gRNAs used, their gene deletion efficiencies, and the specific gene mutations observed are described in Additional file 1: Table S2.

**Fig. 3:**
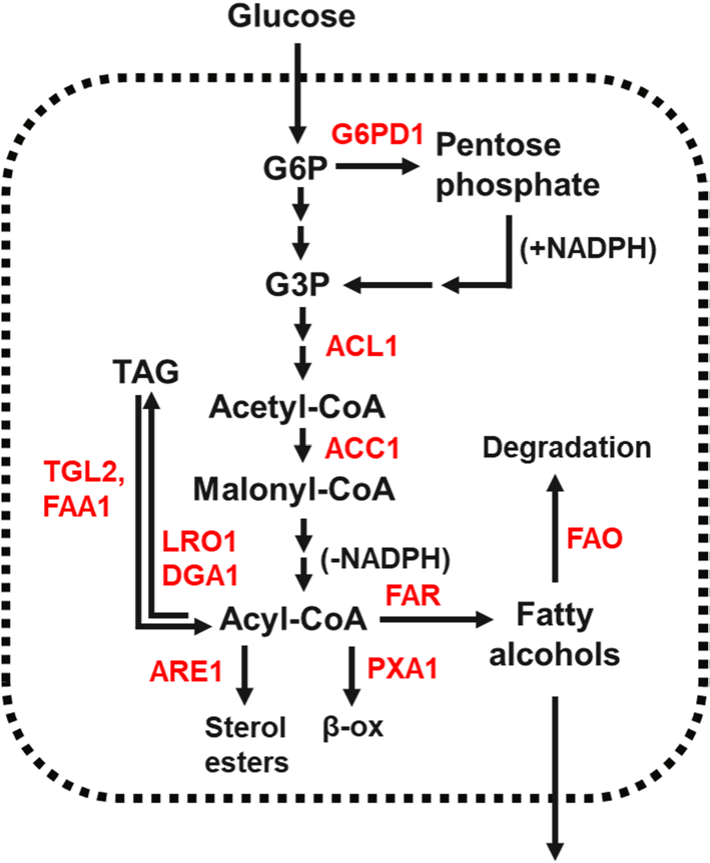
Diagram of the roles metabolic engineering targets explored in this study play within the central carbon metabolism of *R. toruloides*. *G6PD* glucose 6-phosphate dehydrogenase, *ACL1* ATP-citrate lyase, *ACC1* acetyl-CoA carboxylase, *LRO1* lecithin cholesterol acyltransferase, *DGA1* diacylglycerol acyltransferase, *TGL2* triglyceride lipase, *FAA1* fatty acyl-CoA synthetase, *ARE1* acyl-CoA:sterol acyltransferase, *FAR* fatty acyl-CoA reductase, *PXA1* peroxisomal ABC-transporter, *FAO* fatty alcohol oxidase

**Fig. 4:**
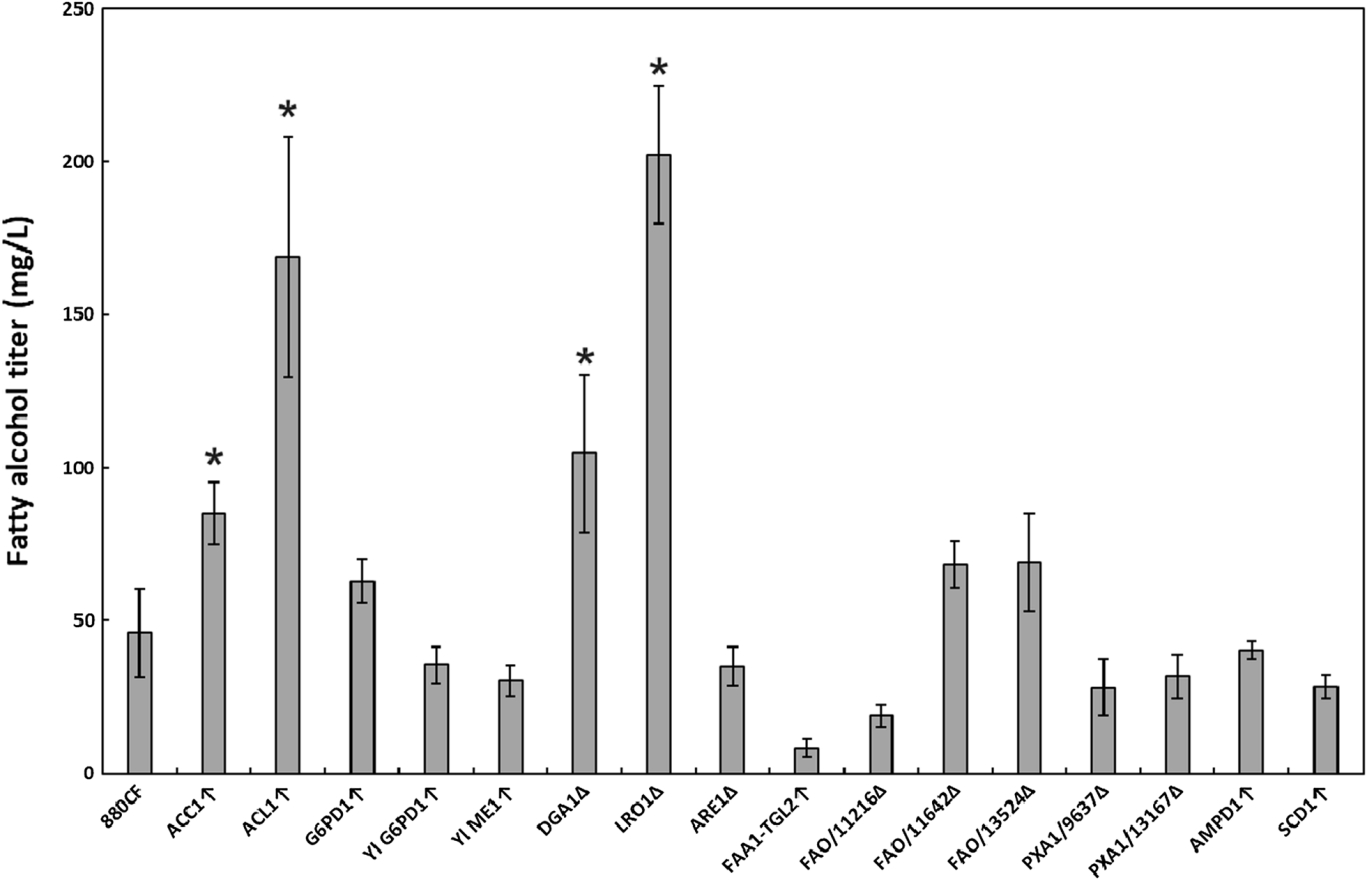
Fatty alcohol titers produced from single mutant strains derived from 880CF and tested in glass culture tube fermentation. For genes with multiple identified homologs, the Mycocosm proteinID is provided. Error bars represent ± standard deviation of biological triplicates. Presence of an asterisk indicates statistically significant improvement compared to 880CF (*p* < 0.05, student *t*-test). *ACC1* acetyl-CoA carboxylase gene, *ACL1* ATP-citrate lyase gene *G6PD1* *R. toruloides* glucose-6-phosphate dehydrogenase gene, *Yl G6PD* *Y. lipolytica* glucose-6-phosphate dehydrogenase gene, *Yl ME1* gene encoding *Y. lipolytica* malic enzyme, *DGA1* diacylglycerol acyltransferase gene, *LRO1* phospholipid:DAG acyltransferase gene, *ARE1* acyl-CoA:cholesterol acyltransferase gene, *FAA1* fatty acyl-CoA synthase gene, *TGL2* triglyceride lipase gene, *FAO* fatty alcohol oxidase gene, *PXA1* peroxisomal ABC transporter gene, *AMPD1* AMP deaminase gene, *SCD1* stearoyl-CoA desaturase gene

Many genetic targets and strategies have been explored for the metabolic engineering of different yeast species for fatty alcohol production. For the engineering of *R. toruloides*, we tested various approaches that have shown prior success in yeast including upregulating the production of precursor metabolites, blocking pathways that compete for metabolic flux, reactivating acyl chains sequestered in lipids, and blocking fatty alcohol degradation pathways [9, 14] (Fig. 3). For overexpression targets, the native genomic copy of a gene was used unless otherwise noted. Mycocosm proteinIDs are provided for each native gene targeted in Additional file 1: Table S3 and a full list of strains created in this study is provided in Additional file 1: Table S4. The strains created were compared in glass culture tubes containing synthetic complete media with a 10% dodecane overlay inoculated to an initial OD600 of 0.1. The fatty alcohol concentration in the overlay was measured by GC-FID after 6 days of growth (Fig. 4).

*Increasing production of precursor metabolites:* MaFAR produces fatty alcohols through the reduction of fatty acyl-CoA chains, consuming two molecules of NADPH per fatty alcohol molecule produced [23]. Fatty acyl-CoA chains are produced by the fatty acid synthase complex, consuming one malonyl-CoA molecule and two NADPH molecules per two-carbon extension of the growing acyl-CoA chain [1]. Therefore, we tested overexpression of the native *ACL1* to increase cytosolic acetyl-CoA production, overexpression of the native *ACC1* to increase malonyl-CoA production from acetyl-CoA, and overexpression of three NADPH-producing genes (*R. toruloides* glucose-6-phosphate dehydrogenase gene (*G6PD1*), *Y. lipolytica G6PD1*, and *Y. lipolytica* malic enzyme gene (*ME1)*).

*ACC1* and *ACL1* overexpression resulted in notable 1.8-fold and 3.7-fold increases in the fatty alcohol titer, respectively. Overexpression of the native *G6PD1* or *Y. lipolytica G6PD1* and *ME1* did not significantly increase the titer.

*Blocking or reversing lipid formation:* In wild-type *R. toruloides*, as in other oleaginous yeasts, the typical destination for fatty acyl-CoA chains is storage in lipid droplets as triacylglycerides (TAGs) [29]. We attempted to block this competing pathway through the disruption of two genes involved in TAG formation, *DGA1* and *LRO1*. *DGA1* catalyzes the transfer of an acyl chain from an acyl-CoA molecule to a diacylglyceride (DAG), while *LRO1* forms TAGs by transferring an acyl chain from a phospholipid to a DAG [30]. We additionally tested the disruption of *ARE1* encoding an acyltransferase involved in sterol esterification [31].

Disruption of *DGA1* and *LRO1* increased the fatty alcohol titer by 2.3-fold and 4.4-fold, respectively, indicating that these acyltransferases (or in the case of *LRO1*, possibly an upstream protein as acyl-CoA is not thought to be a direct substrate) are noteworthy competitors with MaFAR for acyl-CoA molecules. *ARE1* disruption, however, did not increase the fatty alcohol titer. Due to the promising results of *DGA1* and *LRO1* disruption, simultaneous knockout of both genes was attempted by integrating a single construct with expression cassettes for gRNAs targeting both genes and sequencing the resulting colonies at both gene loci. However, only single knockouts, and a small number of very slow-growing colonies unable to be sequenced were obtained, suggesting the *DGA1*Δ*LRO1*Δ double mutation may be lethal, or strongly detrimental to growth in *R. toruloides*. As these two genes are expected to account for the majority of TAG formation [30], one possible explanation for the failure to obtain the double mutant is that the inability to form TAGs confers a severe fitness defect in *R. toruloides*.

An alternative approach we explored was to reactivate acyl chains that have been sequestered in TAGs, first by hydrolyzing TAGs with triglyceride lipase (*TGL2*) to form free fatty acids, then reactivating the free fatty acids with acyl-CoA synthetase (*FAA1*). As overexpression of both genes may be necessary for this strategy to work, *FAA1* and *TGL2* overexpression cassettes driven by the *ANT1* and *TEF1* promoters, respectively, were cloned to a single plasmid and integrated simultaneously. However, overexpression of these two genes did not increase the fatty alcohol titer.

*Blocking fatty alcohol degradation:* The elimination of fatty alcohol degradation pathways has been shown to improve fatty alcohol titers in *S. cerevisiae* and *Y. lipolytica* [9, 14]. Three IFO0880 genes were identified with homology to *Y. lipolytica* fatty alcohol oxidase gene (*FAO1*). Furthermore, a popular strategy for increasing lipid production which has also proved effective in improving fatty alcohol titers is to disrupt β-oxidation, either by disrupting peroxisome biogenesis through deletion of *PEX10* [32, 33], or by interfering with peroxisomal uptake of fatty alcohols or fatty acyl-CoA by deleting the peroxisomal transporter genes *PXA1* and *PXA2* [14]. While *PEX10* is reported to be essential in *R. toruloides* [27] (and supporting this, we were unable to obtain any viable *PEX10*Δ mutants), two genes were identified with homology to *S. cerevisiae PXA1* and *PXA2* and successfully targeted for disruption. However, disruption of the three *FAO* genes and both *PXA1*-like genes did not significantly improve the fatty alcohol titer.

*Other targets:* Overexpression of stearoyl-CoA desaturase gene (*SCD1* or *OLE1*) [34] and AMP deaminase gene (*AMPD1*) [33] have both been shown to increase lipid production in *Y. lipolytica*, while *SCD1* overexpression also improved fatty alcohol production in *S. cerevisiae* [14]. *AMPD1* is involved in the initiation of lipid biosynthesis, reducing the mitochondrial AMP concentration to inhibit isocitrate dehydrogenase, resulting in the accumulation of citrate, which can be converted to cytosolic acetyl-CoA by ACL1 following export from the mitochondria [35]. SCD1 converts stearoyl-CoA to oleyl-CoA, relieving stearoyl-CoA-mediated allosteric inhibition of ACC1, allowing for more malonyl-CoA formation in the presence of long-chain acyl-CoA molecules [34]. However, we found that neither of these approaches increased the fatty alcohol titer in *R. toruloides*.

**Fig. 5:**
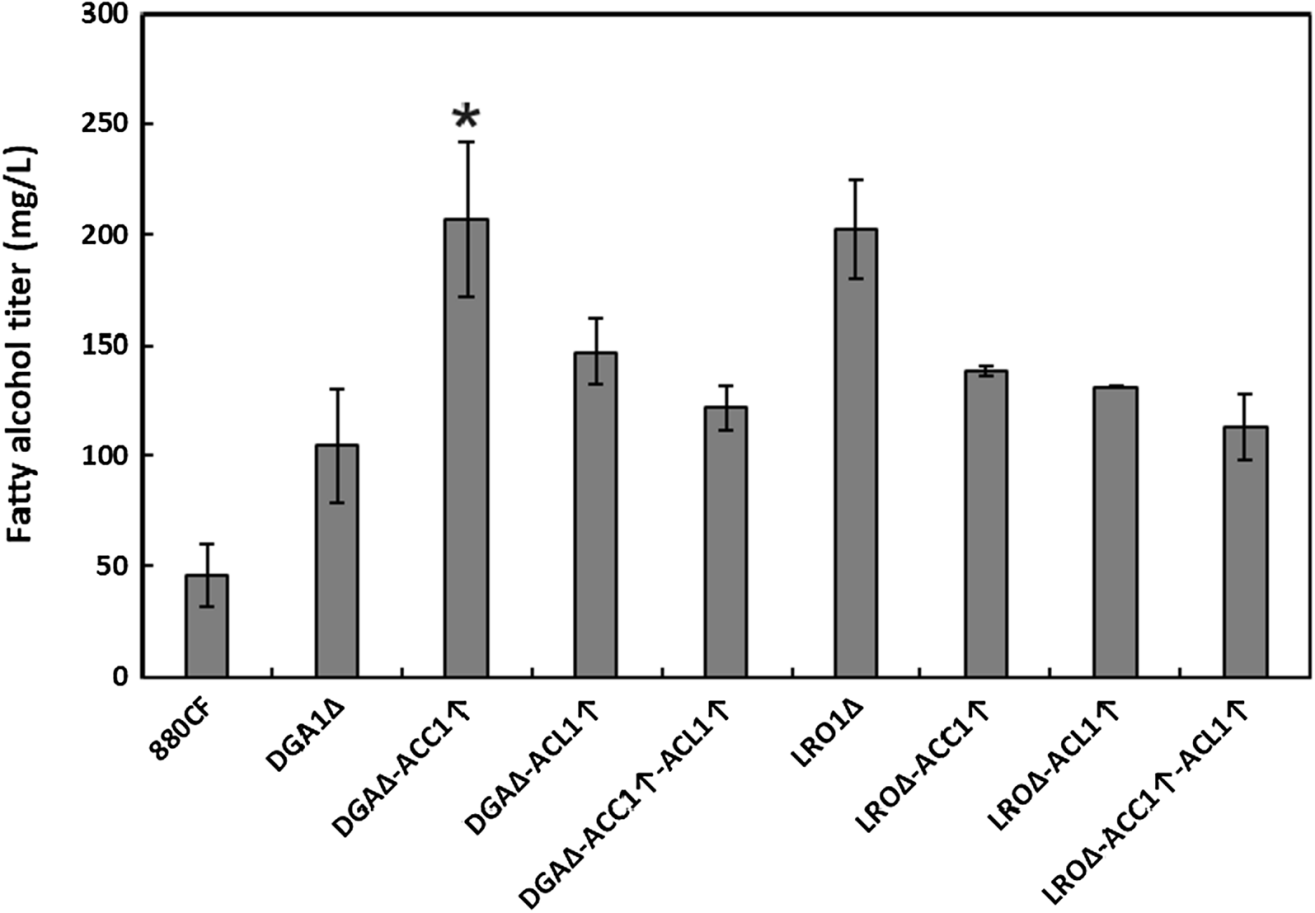
Fatty alcohol titers produced from double and triple mutant strains derived from 880CF and tested in glass culture tube fermentations. Error bars represent ± standard deviation of biological triplicates. Presence of an asterisk indicates statistically significant improvement compared to the parent strain (*p* < 0.05, student *t*-test). *ACC1* acetyl-CoA carboxylase gene, *ACL1* ATP-citrate lyase gene, *DGA1* diacylglycerol acyltransferase gene, *LRO1* phospholipid:DAG acyltransferase gene

*Combinatorial exploration of validated single targets:* Although we were unable to disrupt the two most promising deletion targets, *LRO1* and *DGA1*, in the same strain, both single knockouts were evaluated in combination with the two best overexpression targets, *ACC1* and *ACL1*, individually and in tandem (Fig. 5). Combination of *DGA1*Δ with *ACC1* overexpression was found to increase the fatty alcohol titer from the initial 106 mg/L to 206 mg/L. However, all of the *LRO1*Δ strains with *ACC1* or *ACL1* overexpression had lower fatty alcohol titers than the starting strain’s titer of 202 mg/L.

#### Bioreactor fermentation of fatty alcohols

**Fig. 6:**
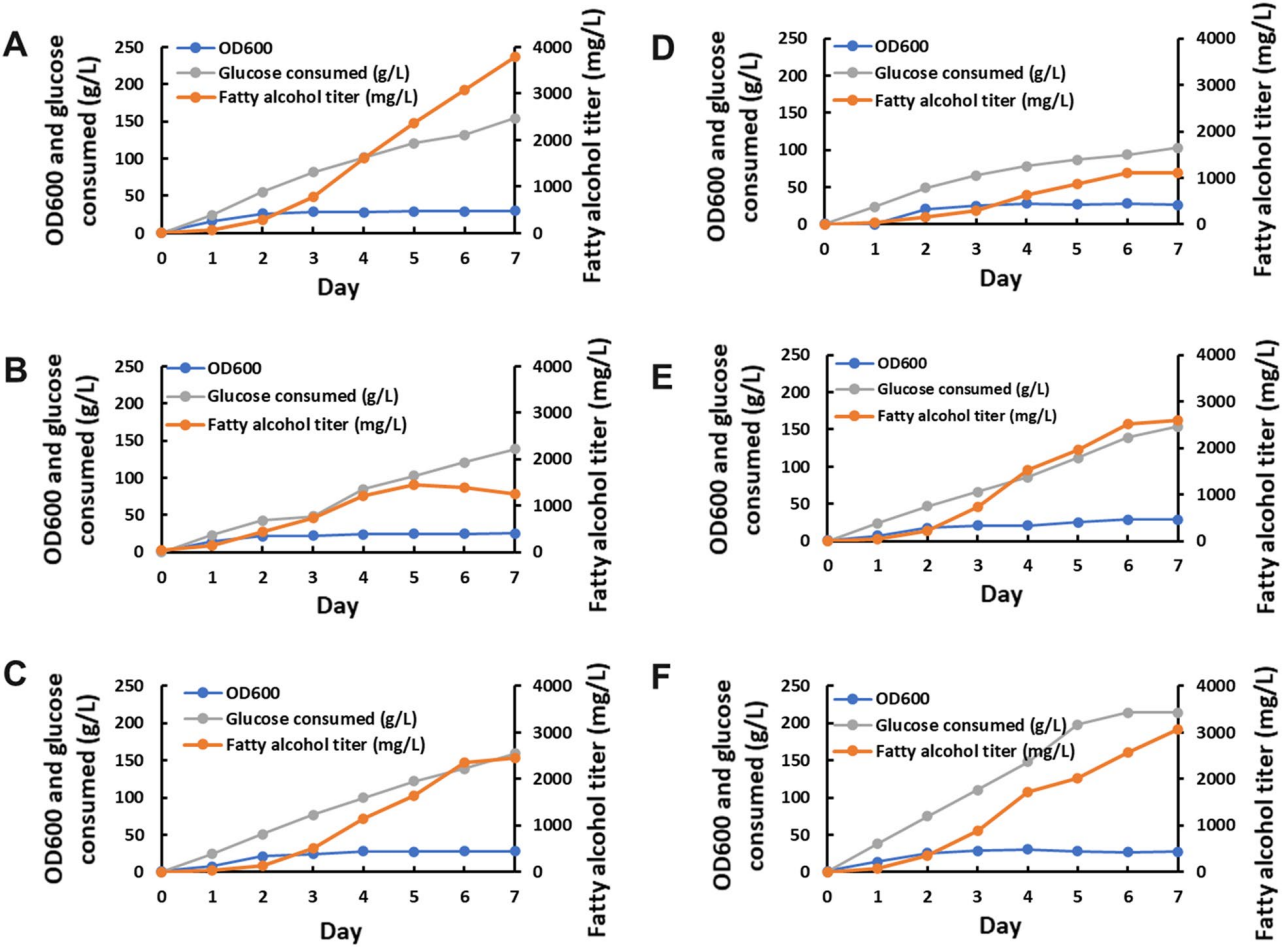
Fatty alcohol titers produced, glucose consumed, and OD600 values in bioreactor fermentations of the strains **A** 880CF-*LRO1*Δ, **B** 880CF-*LRO1*Δ-*ACL1*, **C** 880CF-*LRO1*Δ-*ACC1-ACL1*, **D** 880CF-*DGA1*Δ, **E** 880CF-*DGA1*Δ-*ACC1*, and **F** 880CF-*DGA1*Δ-*ACC1-ACL1*

Three of the best-performing *DGA1*Δ-based strains (*DGA1*Δ, *DGA1*Δ-*ACC1*, and *DGA1*Δ-*ACC1-ACL1*) and *LRO1*Δ-based strains (*LRO1*Δ, *LRO1*Δ-*ACL1*, and *LRO1*Δ-*ACC1-ACL1*) were tested in 250 mL Eppendorf DASbox Mini Bioreactors with a 20% dodecane overlay in week-long fed-batch fermentations with glucose concentrations restored to 50 g/L each day for the first five days (Fig. 6, Table 2). As in the culture tube-scale tests, the *LRO1*Δ strain performed better than its derivatives with additional overexpression targets, while the *DGA1*Δ strain benefitted from *ACC1* and *ACC1-ACL1* overexpression. The highest fatty alcohol titer and yield of 3.7 g/L and 0.024 g/g glucose, respectively, were obtained in 880CF-*LRO1*Δ. In each experiment, the peak fatty alcohol titer was reached no later than day 7. Each strain showed similar growth characteristics, reaching an OD600 between 25 and 30 after approximately 48 h of growth. In most cases, major fatty alcohol secretion began at this point and continued until ~ 48 h after the glucose feed was stopped.

**Table 2:** Peak fatty alcohol titers and yields resulting from pulse-fed batch fermentation in Eppendorf DASbox Mini Bioreactors for 7 days

Genotype	FOH titer (mg/L)	FOH yield (g/g glucose)
880CF-*LRO1*Δ	3.7	0.024
880CF-*LRO1*Δ-*ACL1*	1.5	0.014
880CF-*LRO1*Δ-*ACC1*-*ACL1*	2.7	0.016
880CF-*DGA1*Δ	1.1	0.011
880CF-*DGA1*Δ-*ACC1*	2.7	0.017
880CF-*DGA1*Δ-*ACC1*-*ACL1*	3.1	0.014

### Lipidomic analysis of *DGA1*Δ and *LRO1*Δ *Rhodotorula* strains

Our finding that *LRO1*Δ improves fatty alcohol production more than *DGA1*Δ in *R. toruloides* stands in contrast to a report in *S. cerevisiae*, where *DGA1* deletion was highly beneficial but *LRO1* deletion abolished fatty alcohol production entirely [14]. It is surprising that *LRO1*Δ would improve fatty alcohol production so significantly, as the reaction LRO1 is known to catalyze (reversible transfer of an acyl chain from a phospholipid to a DAG) does not directly use acyl-CoA as a substrate, unlike DGA1 which consumes acyl-CoA to convert DAGs into TAGs.

**Fig. 7:**
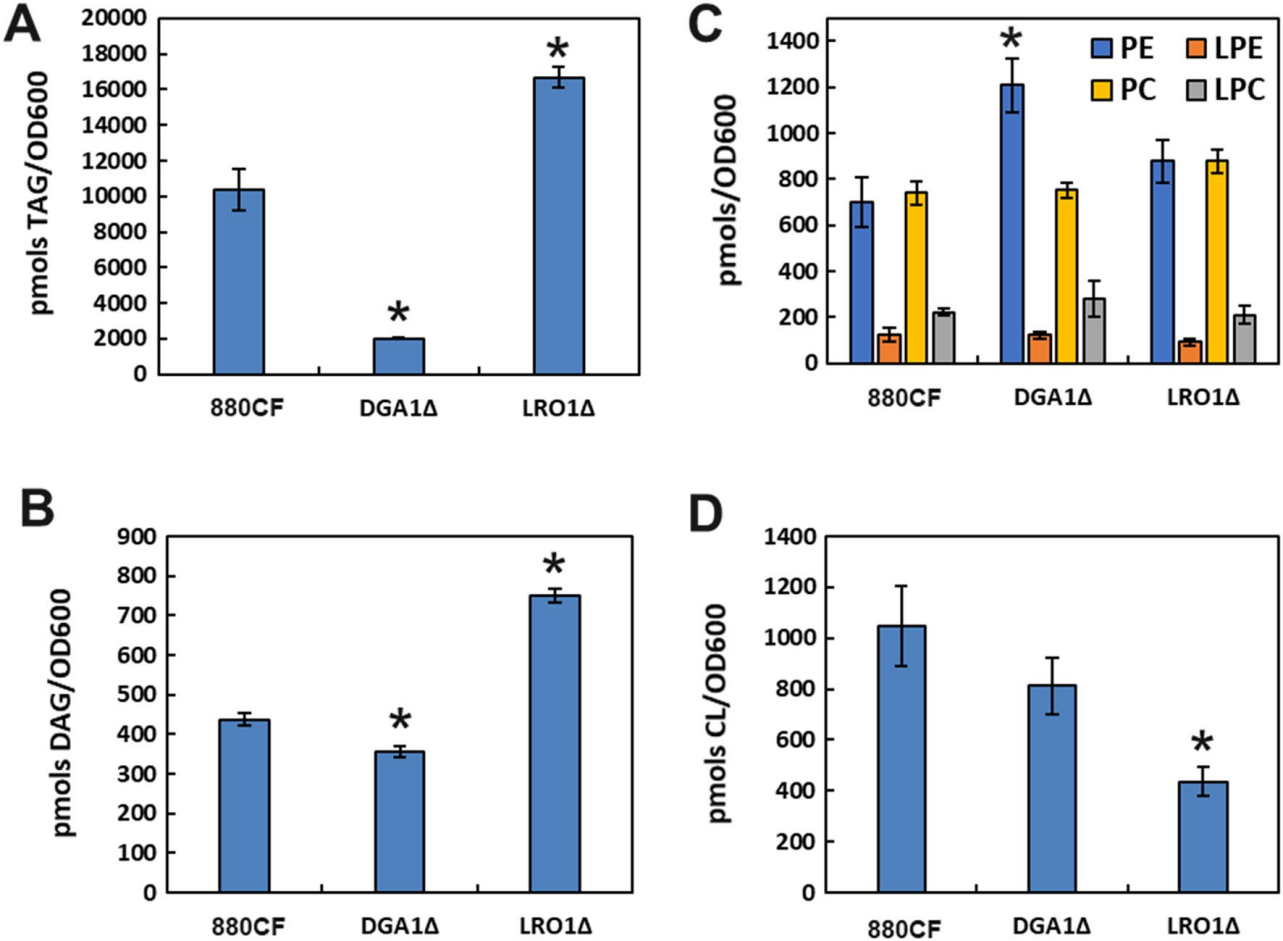
Intracellular concentrations of the lipid species **A** TAG; **B** DAG; **C** PC, PE, LPC and LPE; and **D** CL as determined by lipidomics, in the strains 880CF, 880CF-*DGA1*Δ, and 880CF-*LRO1*Δ. Error bars represent ± standard deviation of biological triplicates. *PE* phosphatidylethanolamine, *PC* phosphatidylcholine, *LPC* lyso-phosphatidylcholine, *DAG* diacylglyceride, *TAG* triacylglyceride, *LPE* lyso-phosphatidylethanolamine, *CL* cardiolipin. Presence of an asterisk indicates statistically significant improvement compared to 880CF (*p* < 0.05, student *t*-test)

To investigate and compare the mechanisms by which these knockouts lead to improved fatty alcohol production in *R. toruloides*, a lipidomic study was performed comparing the strains 880CF, 880CF-*LRO1*Δ, and 880CF-*DGA1*Δ after six days of growth on synthetic media. Most strikingly, the *DGA1*Δ mutant has greatly reduced TAG accumulation, as expected, while the *LRO1*Δ mutant has increased TAG and DAG accumulation (Fig. 7a, b). This result suggests that DGA1 is likely a net TAG producer and direct competitor with MaFAR for acyl-CoA molecules as expected, while either LRO1 is a net TAG consumer under our experimental conditions, moving acyl chains from the acylglycerol population to lyso-phospholipids, or the deletion of *LRO1* has increased the overall carbon flux entering lipid biosynthesis.

No shifts in the balances between phosphatidylcholine (PC) and lyso-PC (LPC), or phosphatidylethanolamine (PE) and lyso-PE (LPE) were observed (Fig. 7c), and no other major lipid species were significantly depleted in the *LRO1*Δ mutant (Additional file 1: Table S5) suggesting the extra carbon entering the DAG and TAG pools in this mutant must have come from outside the lipid network. A comparison of the carbon consumption of the three strains showed 880CF-*LRO1*Δ having the highest glucose consumption after six days of growth, at 14 g/L compared to 10.5 g/L consumed by 880CF (Additional file 1: Fig. S1). Therefore, we consider the most likely explanation for the increased fatty alcohol production and DAG/TAG production of 880CF-*LRO1*Δ to be a general increase in sugar uptake and carbon flux entering the lipid biosynthesis pathway, although the exact mechanism causing this increase in flux warrants further study. Finally, we also observed that cardiolipin (CL) levels in the *LRO1*Δ strain were significantly lowered (Fig. 7d), suggesting disruptions to phosphatidylglycerol metabolism may have occurred as a result of the knockout, although the decrease in this membrane lipid species alone is not enough to account for the much larger increase in TAGs and DAGs.

## Discussion

While *R. toruloides* has been explored as a production host for a variety of molecules, fewer efforts have aimed to engineer its metabolism and improve product titers, particularly with strategies incorporating the still-difficult process of gene disruption. Zhang and coworkers identified a number of overexpression targets capable of improving lipid production in *R. toruloides*, including *ACC1*, *DGA1*, *ME1*, and *SCD1,* and investigated the knockout of a single gene, *PEX10*, although its knockout did not increase the lipid titer [12, 25]. However, numerous *R. toruloides* strains developed to produce other bioproducts such as terpenoids [36–38], fatty acid ethyl esters [39], and, until this study, fatty alcohols [10, 22] have not been optimized further than the expression of the heterologous enzyme responsible for the formation of the final product. Therefore, the development of a workflow for performing sophisticated metabolic engineering as well as the identification of broadly useful metabolic engineering targets in this non-model yeast is of significant importance to the research community.

In this study, a CRISPR/Cas9 tool based on previous reports [15, 16] was used in conjunction with constitutive gene overexpression to create an array of single, double, and triple overexpression/deletion mutants in a fatty alcohol-producing strain derived from IFO0880. Notably, the use of CRISPR/Cas9 for the generation of knockouts greatly improved the throughput of the *R. toruloides* strain engineering process, allowing the efficient creation of eight knockout strains for characterization. In screening the engineered strains, we found that increasing the supply of precursor metabolites in the form of acetyl-CoA and malonyl-CoA through overexpression of *ACL1* and *ACC1* and reducing competition for fatty acyl-CoA molecules from the lipid biosynthetic pathway through disruption of *DGA1* and *LRO1* proved to be the most effective strategies for improving the fatty alcohol titer. Increasing the production of the reducing cofactor NADPH, used for acyl-CoA biosynthesis and the reduction of acyl chains to fatty alcohols, was less effective, suggesting NADPH supply may not be limiting for this product in *R. toruloides*. Disruption fatty alcohol oxidase-like genes (identified based on homology to *Y. lipolytica FAO1*) and peroxisomal transporter *PXA1*-like genes had no effect, as did overexpression of *SCD1, AMPD1*, and *FAA1* + *TGL2*, and disruption of *ARE1*.

While *LRO1* and *DGA1* were selected as knockout targets based on similar reasoning (attempting to increase availability of acyl-CoA chains by preventing their incorporation into TAGs), the significantly differing properties of the resulting knockout strains highlight the different metabolic roles played by these enzymes in *R. toruloides*. Most notably, while the *DGA1*Δ mutant had largely abolished TAG formation compared to the wild-type strain, the *LRO1*Δ mutant surprisingly had elevated DAG and TAG levels. This stands in contrast to *Y. lipolytica*, where *DGA1*Δ and *LRO1*Δ were found to moderately lower TAG levels individually and a *DGA1*Δ *LRO1*Δ double mutant showed a more substantial decrease in TAG [30]. An additional report in *Y. lipolytica* demonstrated that in this yeast species, *overexpression* of *LRO1* increased lipid production [40]. Our finding in *R. toruloides* implicates *DGA1* as the major producer of TAGs, while the role of *LRO1* is less certain. For future studies of lipid production in *R. toruloides*, both overexpression and knockout of *LRO1* may be informative to explore. The fact that the *LRO1*Δ mutant showed a greater improvement in fatty alcohol production than the *DGA1*Δ mutant is partially explained by the increased glucose consumption of the *LRO1*Δ mutant, although the *LRO1*Δ mutant also showed a higher carbon yield in culture tube and bioreactor fermentations.

Combining the beneficial overexpression of *ACL1* and *ACC1* was found to further increase the fatty alcohol titer in the *DGA1*Δ strain background, up to 3.1 g/L in bioreactor fermentation, while the *LRO1*Δ mutant’s titer of 3.7 g/L in the bioreactor was not further improved. This discrepancy may be due to different characteristics of the starting strains, with 880CF-*DGA1*Δ producing about half as much fatty alcohol as 880CF-*LRO1*Δ. As both of these knockouts are reasoned to improve the fatty alcohol production by redirecting fatty acyl-CoA flux from TAG formation to fatty alcohol formation, 880CF-*LRO1*Δ likely has a higher level of fatty acyl-CoA available as substrate for the FAR. Overexpression of *ACC1* and *ACL1* is intended to increase production of fatty acyl-CoA, but availability of this precursor may have been a limiting factor for fatty alcohol production only in 880CF-*DGA1*Δ.

Cordova and coworkers were able to produce 5.8 g/L fatty alcohols in a *Y. lipolytica* strain lacking β-oxidation (*MFE1*Δ) and peroxisome biogenesis (*PEX10*Δ), but containing *DGA1* overexpression [9]. While disruption of peroxisomal transporters was ineffective in our study and a *PEX10*Δ mutant could not be obtained (the gene has been reported to be essential in IFO0880 [27]), in the future a more comprehensive elimination of β-oxidation, such as by deletion of *MFE1*, may further improve fatty alcohol production. The high fatty alcohol titer in a *DGA1*-overexpressing *Y. lipolytica* strain is interesting, but it may be that *DGA1*Δ rather than overexpression would further improve the titer of fatty alcohols as strains with native *DGA1* expression levels or *DGA1*Δ were not compared. In our study and D’Espaux and coworkers’ study in *S. cerevisiae*, *DGA1*Δ improved fatty alcohol production [14]. Also in agreement between these studies is the beneficial effect of overexpression of *ACC1*, while upregulation of unsaturated fatty-acyl production to potentially relieve feedback inhibition of saturated fatty acyl-CoA on the fatty acid synthase complex [1] was more beneficial in *S. cerevisiae* (*OLE1* overexpression) than *R. toruloides* (*SCD1* overexpression), resulting in a fourfold increase in fatty alcohol titer compared to the parent strain. In contrast, *LRO1*Δ was effective in promoting *R. toruloides*’s fatty alcohol production but detrimental to *S. cerevisiae*’s. The difference in response to *SCD1* overexpression may be attributable to the different fatty acid profiles of *S. cerevisiae* and *R. toruloides*: *S. cerevisiae* produces predominantly saturated C16 fatty acids and alcohols, while saturated and unsaturated C18 fatty acids and alcohols are major components of the *R. toruloides* product spectrum [10, 14]. This difference could confound the effect of *SCD1* overexpression in numerous ways, such as through additional post-translational regulation of the SCD1 protein, or through the presence of much greater amounts of saturated C18-CoA substrate for the enzyme to act on to meaningfully shift the fatty acid profile.

Production of fatty alcohols has been previously reported in *R. toruloides* through heterologous expression of MaFAR without further engineering, with 8 g/L achieved in a bioreactor on YP-sucrose with 5 g/L tergitol and no organic overlay [10] and 1.7 g/L achieved in a bioreactor on synthetic complete media with 0.1 g/L of the nonionic surfactant tergitol and a 20% vol/vol dodecane overlay to improve fatty alcohol extraction [22]. This study, in comparison, achieved a maximum titer of 3.7 g/L in a bioreactor on synthetic complete media without tergitol and with a 20% vol/vol dodecane overlay in a *LRO1*Δ strain. Improved titers were also identified in *DGA1*Δ as well as *ACC1* and *ACL1* overexpressing strains identifying all of these targets as well as *LRO1*Δ as relevant to the fatty alcohol overproduction phenotype in *R. toruloides*. Combination of these metabolic engineering targets with other strategies such as addition of nonionic surfactants or different media formulations may enable additional increases in fatty alcohol productivity from *R. toruloides*. Furthermore, as the gene editing toolkit for *R. toruloides* becomes more advanced, the use of selection marker recycling or an episomal plasmid will facilitate the creation of strains with a larger number of modifications and further improved titers.

## Conclusions

In this study, a Cas9-FAR-expressing strain of *R. toruloides* IFO0880 was created and used as a platform organism for the exploration of 16 overexpression and deletion metabolic engineering targets. Several promising targets were explored combinatorially at the culture tube and bioreactor scale, and the best-performing strain, harboring *LRO1*Δ, produced 3.7 g/L fatty alcohols from synthetic media. In contrast to findings in other yeast, *DGA1* and *LRO1* knockouts were both found to increase fatty alcohol production in *R. toruloides*, while a lipidomic survey showed that DGA1 is the predominant TAG-producing protein in this yeast. Based on these findings, the role of *LRO1* in *R. toruloides* lipid biosynthesis and fatty acyl-CoA consumption appears to differ from other yeasts. Further investigation may lead to new insights about the lipid metabolism of *R. toruloides*.

## Methods

### Strains and media

The *R. toruloides* strain IFO0880 was grown at 30 °C, 250 rpm. YPD media (10 g/L yeast extract, 20 g/L peptone, 20 g/L glucose) was used for routine handling of cells. For selection or maintenance of transformants, 200 µg/mL G418 (KSE Scientific, Durham, NC), 100 µg/mL nourseothricin (Gold Biotechnology, St. Louis, MO), or 50 µg/mL hygromycin (Gold Biotechnology, St. Louis, MO) was supplemented. Fatty alcohol production was measured in synthetic complete medium (1.7 g/L yeast nitrogen base (BD, Franklin, NJ), 5 g/L ammonium sulfate, 0.78 g/L complete synthetic mixture (MP Biomedicals, Santa Ana, CA), 40 g/L glucose) adjusted to pH 7 using sodium hydroxide. Culture tube-scale fatty alcohol fermentation experiments were performed by preculturing the yeast for 48 h in SC media, then inoculating 5 mL of SC media in glass culture tubes to an OD600 of 0.1, with a 10% dodecane overlay containing 100 mg/L of pentadecane as internal standard and grown for 6 days.

Standard cloning was performed in the *Escherichia coli* strain NEB10β (New England Biolabs, Ipswich, MA). Cells were grown on Luria Broth (LB) medium at 37 °C, 250 rpm with 100 µg/mL ampicillin or 50 µg/mL kanamycin. Multi-fragment cloning of 20 kb and larger plasmids was performed in *S. cerevisiae* BY4741 using DNA assembler [41], with growth on YPD or synthetic uracil dropout media (1.7 g/L yeast nitrogen base, 5 g/L ammonium sulfate, 0.78 g/L complete synthetic mixture without uracil, 20 g/L glucose).

### Gene synthesis

Genes were synthesized by Twist Bioscience (San Francisco, CA) following codon optimization by the JGI BOOST tool, set to use the most frequent codon for each amino acid as they occur in *R. toruloides* [42].

### Plasmid construction

The plasmid pRTN was constructed for heterologous expression or native gene overexpression from the *E. coli* elements of pUC19 (pMB1 origin, ampicillin resistance), the *S. cerevisiae* elements of pRS426 (2µ origin and *URA3* selection marker), the strong *R. toruloides* p17 promoter, GFP, the T35S terminator, and a *R. toruloides* nourseothricin resistance cassette from pGI2 [25] using DNA assembler. The promoter was subsequently replaced if needed using *Afl*II and *Mfe*I digestion, and expressed gene was replaced using *Mfe*I and *Spe*I digestion. Promoters and native genes were PCR amplified from IFO0880 genomic DNA extracted using lithium acetate/SDS/heat lysis [43], while heterologous genes were synthesized by Twist Bioscience. Multi-gene expression plasmids were pieced together from either the pRTN or NM9 [15] backbones (for nourseothricin or G418 resistance, respectively) and the appropriate promoter and gene elements using DNA assembler. To prevent complications in the assembly, unique terminators (Tnos, T35S, and Ttub) were used for each gene and the selection marker.

The plasmid pRTH was constructed for gRNA cloning and expression from the *E. coli* elements of pUC19 (pMB1 origin, ampicillin resistance), the *S. cerevisiae* elements of pRS426 (2µ origin and *URA3*), a gRNA expression cassette with the IFO0880 5S rRNA, tRNA^Tyr^, 2 *Bsa*I sites, the *S. cerevisiae* SUP4 terminator, and a *R. toruloides* hygromycin resistance cassette from pZPK-PGPD-HYG-Tnos [44] using DNA assembler. gRNAs were cloned by digesting pRTH with *Bsa*I and ligating two annealed and 5’-phosphorylated oligos with a forward strand 5’ GGGA and reverse strand 5’ AAAC overhang. For attempted double deletions, 2 tandem gRNA expression cassettes were synthesized as a gBLOCK and cloned into the same *Bsa*I restriction sites using Golden Gate assembly. A list of primers used in this study is provided in Additional file 1: Table S6, and a list of plasmids created is provided in Additional file 1: Table S7.

### Transformation of *R. toruloides*

*R. toruloides* was transformed using heat shock as has been described previously [16]. Briefly, a colony was picked and cultured overnight in YPD supplemented with an appropriate antibiotic if necessary. The overnight culture was used to inoculate a shake flask with 25 or 50 mL of YPD (for up to 5 or 10 transformations, respectively) to an OD600 of 0.2 and cultured for 4 h. The cells were collected by centrifugation, washed with water twice, and mixed with 240 µL PEG3350 (Sigma Aldrich, St. Louis, MO), 36 µL 1 M lithium acetate (Sigma Aldrich, St. Louis, MO), 50 µL of 2 mg/mL salmon sperm DNA (Sigma Aldrich, St. Louis, MO), and 1–2 µg of linear DNA dissolved in 40 µL of water. The cells were incubated with shaking in the transformation mixture for 30 min at 30 °C. Then, 34 µL of DMSO was added to the mixture, which was briefly vortexed, and heat shocked at 42 °C for 15 min. The cells were collected, washed once with water, resuspended in 2 mL YPD, and allowed to recover overnight. The cells were then collected and spread to YPD agar plates supplemented with the appropriate antibiotic. Transformation efficiencies of ~ 10^2^ CFU/µg DNA were typically observed.

### Genetic manipulation of *R. toruloides*

All genetic modifications to *R. toruloides* were made by random integration of linear PCR or restriction digestion fragments using heat shock transformation. After cloning of the gene or gRNA expression cassette to be transformed to a plasmid as described above, fragments were prepared either through PCR amplification (with the primers ZPK F/R, or gRNA F/R, respectively) from their plasmid, or (for cassettes longer than 7 kb) through excision of this fragment by digestion with suitable restriction enzymes, followed by spin column purification. Overexpression cassettes were created by expressing either the *R. toruloides* genomic copy of a gene (for endogenous genes) or a synthetic codon optimized gene (for heterologous genes) with the strong *p17* promoter. Deletion mutants were created by transforming a single gRNA targeting the first 10% of the target gene ORF to generate frame shift mutations following NHEJ DNA repair. gRNAs were designed using the Benchling gRNA tool. For verification of deletion mutants, genomic DNA was extracted using lithium acetate/SDS/heat lysis [43], PCR amplified at the target locus and sequenced. For gene activation targets, genomic DNA was extracted using the same method and integration was verified using colony PCR.

### Bioreactor fermentation

For bioreactor experiments, a seed culture was grown for 48 h in a culture tube in SC media, then transferred entirely to 50 mL of media in a shake flask for another 24 h. The cells were collected, washed once with water, and used to inoculate 100 mL of SC media to an OD600 of 1 in 250 mL Eppendorf DASbox Mini Bioreactors (Eppendorf, Hamburg, Germany) with 20 mL of dodecane containing 100 mg/L pentadecane. Bioreactors were set to maintain a pH of 7, agitation of 1200 rpm, temperature of 30 °C, and air flow rate of 6 standard liters per minute. Antifoam 204 was added to control foaming as necessary (typically 1 drop per reactor). Each day, samples were collected to measure OD600, fatty alcohol titer, and glucose concentration. Glucose was added to restore the concentration to 50 g/L for the first five days.

## Analytical methods

Culture tube and bioreactor fermentation experiments were performed with a 10% or 20% dodecane overlay, respectively, with 100 mg/L pentadecane added as an internal standard. For quantification of fatty alcohols, 200 µL or 1 mL (for culture tube and bioreactor, respectively) was removed and centrifuged to separate the dodecane layer. 10 µL of dodecane was mixed with 90 µL of ethyl acetate and analyzed on an Agilent 8860 GC-FID with a 30-m DB-5 column (Agilent, Santa Clara, CA) with a temperature ramp of 70 °C for 3 min, increase at 20 °C/minute to 320 °C, hold 320 °C for 1 min. The total fatty alcohol titer was calculated as the sum of 1-hexadecanol, 1-octadecanol, and oleyl alcohol titers.

Glucose consumption was measured using an Agilent 1260 Infinity HPLC with a refractive index detector (RID) and H^+^ column (Rezex ROA-Organic Acid; Phenomenex, Torrance, CA). The column and detector were run at 50 °C and 0.6 mL/min of 0.005 N H_2_SO_4_ was used as the mobile phase.

### Lipidomics

Cells were cultured in pH7-adjusted SCD media with a 10% dodecane overlay in glass culture tubes under the same conditions as for the fatty alcohol fermentations and cell pellets were collected after 6 days of growth following inoculation. Lipidomics analysis of both the reference and mutant yeast strains was performed using a two-step chloroform–methanol extraction as described elsewhere [45, 46]. Briefly, harvested cells were washed with 150 mM ammonium bicarbonate (ABC) followed by cell lysis with zirconium glass beads. Lipids were extracted from 1 OD unit of cell lysate, to which an internal lipid standard mix was added, in a 2-step method. First extraction was performed with 1 mL of 15:1 chloroform–methanol after which the chloroform layer was aspirated, dried, and then resuspended in an infusion solvent. The second extraction was performed on the remaining aqueous layer with 1 mL of 2:1 chloroform–methanol and again the chloroform layer was aspirated, dried, and resuspended in an infusion solvent. Both resuspended extracts were then infused into a Q-Exactive mass spectrometer via the Advion Triversa Nanomate in the nano-electrospray mode. The infusion solvent used for the dried lipid extract from the 15:1 extraction contained 7.5 mM ammonium formate in a mix of chloroform–methanol–propanol 1:2:4 (v/v) whereas the infusion solvent for the dried extract from the 2:1 extraction contained 0.05% methylamine in a mix of chloroform–methanol 1:5 (v/v) [46].

Identification and quantification of lipid species and classes from the lipidomic data generated by the mass spectrometer was performed using an in-house Python script that employed the pymzml library. Raw data files were input to the pipeline after conversion to the open source mzML format. The data analysis pipeline consisted of scan averaging, offline calibration, deisotoping and identification, quantification, and quantification across replicates. The list of internal standards and their absolute amounts used for spike-in are listed in Additional file 1: Table S8. The code used for analysis is available upon request.

## Supplementary Information

**Additional file 1. Figure S1.** Glucose consumption of 880CF, 880CF-DGA1Δ, and 880CF-LRO1Δ. **Table S1.** DNA sequences of heterologous, codon-optimized genes evaluated in this study. **Table S2.** List of gRNAs used in this study. **Table S3.** List of Mycocosm proteinIDs for R. toruloides IFO0880 gene targets in this study. **Table S4.** List of R. toruloides strains used in this study. **Table S5.** Results of lipidomic comparison of 880CF, 880CF-DGA1Δ, and 880CF-LRO1Δ. **Table S6.** List of primers used in this study. **Table S7.** List of plasmids used in this study. **Table S8.** List of lipidomics internal standards and their absolute amounts used for spike-in.

## Data Availability

All data and materials produced in this study are available upon reasonable request.
