# Supplementary material for: Metabolic engineering of Rhodotorula toruloides IFO0880 improves C16 and C18 fatty alcohol production from synthetic media
Source: Microb Cell Fact. 2022 Feb 19;21:26. doi: 10.1186/s12934-022-01750-3 (PMC8858515; doi:10.1186/s12934-022-01750-3)
Supplement: Supplementary file 1 — Additional file 1. Figure S1. Glucose consumption of 880CF, 880CF-DGA1Δ, and 880CF-LRO1Δ. Table S1. DNA sequences of heterologous, codon-optimized genes evaluated in this study. Table S2. List of gRNAs used in this study. Table S3. List of Mycocosm proteinIDs for R. toruloides IFO0880 gene targets in this study. Table S4. List of R. toruloides strains used in this study. Table S5. Results of lipidomic comparison of 880CF, 880CF-DGA1Δ, and 880CF-LRO1Δ. Table S6. List of primers used in this study. Table S7. List of plasmids used in this study. Table S8. List of lipidomics internal standards and their absolute amounts used for spike-in. [file 12934_2022_1750_MOESM1_ESM.docx]

**Metabolic Engineering of *Rhodotorula toruloides* IFO0880 Improves C16 and C18 Fatty Alcohol Production from Synthetic Media**

J. Carl Schultz^1,2^, Shekhar Mishra^1,2^, Emily Gaither^1,2^, Andrea Mejia^1^, Hoang Dinh^2,3^, Costas Maranas^2,3^, Huimin Zhao^1,2,^*

^1^Department of Chemical and Biomolecular Engineering, Carl R. Woese Institute for Genomic Biology, University of Illinois at Urbana-Champaign, Urbana, IL 61801, United States

^2^U.S. Department of Energy Center for Bioenergy and Bioproducts Innovation (CABBI)

^3^Department of Chemical Engineering, Pennsylvania State University, University Park, PA 16802, United States

^4^Departments of Chemistry, Biochemistry, and Bioengineering, University of Illinois at Urbana-Champaign, Urbana, IL 61801, United States

* To whom correspondence should be addressed. Phone: (217) 333-2631. Fax: (217) 333-5052. E-mail: zhao5@illinois.edu.

**Additional Information**

Figure S1. Glucose consumption of 880CF, 880CF-*DGA1*Δ, and 880CF-*LRO1*Δ

Table S1. DNA sequences of heterologous, codon-optimized genes evaluated in this study

Table S2. List of gRNAs used in this study

Table S3. List of Mycocosm proteinIDs for *R. toruloides* IFO0880 gene targets in this study

Table S4. List of *R. toruloides* strains used in this study

Table S5. Results of lipidomic comparison of 880CF, 880CF-*DGA1*Δ, and 880CF-*LRO1*Δ

Table S6. List of primers used in this study

Table S7. List of plasmids used in this study.

Table S8. List of lipidomics internal standards and their absolute amounts used for spike-in

**Figure S1**. Glucose consumption of 880CF, 880CF-*DGA1*Δ, and 880CF-*LRO1*Δ in glass culture tube growth on pH 7-adjusted SC media with 4% glucose, following inoculation to an initial OD of 0.1. Error bars represent ± standard deviation of biological triplicates.

**Table S1.** DNA sequences of heterologous, codon-optimized genes evaluated in this study.

| **Gene** | **Sequence** |
| --- | --- |
| *MaFAR (maqu_2220)* | ATGGCCATCCAGCAGGTCCACCACGCCGACACCTCGTCGTCGAAGGTCCTCGGCCAGCTCCGCGGCAAGCGCGTCCTCATCACCGGCACCACCGGCTTCCTCGGCAAGGTCGTCCTCGAGCGCCTCATCCGCGCCGTCCCGGACATCGGCGCCATCTACCTCCTCATCCGCGGCAACAAGCGCCACCCGGACGCCCGCTCGCGCTTCCTCGAGGAGATCGCCACCTCGTCGGTCTTCGACCGCCTCCGCGAGGCCGACTCGGAGGGCTTCGACGCCTTCCTCGAGGAGCGCATCCACTGCGTCACCGGCGAGGTCACCGAGGCCGGCTTCGGCATCGGCCAGGAGGACTACCGCAAGCTCGCCACCGAGCTCGACGCCGTCATCAACTCGGCCGCCTCGGTCAACTTCCGCGAGGAGCTCGACAAGGCCCTCGCCATCAACACCCTCTGCCTCCGCAACATCGCCGGCATGGTCGACCTCAACCCGAAGCTCGCCGTCCTCCAGGTCTCGACCTGCTACGTCAACGGCATGAACTCGGGCCAGGTCACCGAGTCGGTCATCAAGCCGGCCGGCGAGGCCGTCCCGCGCTCGCCGGACGGCTTCTACGAGATCGAGGAGCTCGTCCGCCTCCTCCAGGACAAGATCGAGGACGTCCAGGCCCGCTACTCGGGCAAGGTCCTCGAGCGCAAGCTCGTCGACCTCGGCATCCGCGAGGCCAACCGCTACGGCTGGTCGGACACCTACACCTTCACCAAGTGGCTCGGCGAGCAGCTCCTCATGAAGGCCCTCAACGGCCGCACCCTCACCATCCTCCGCCCGTCGATCATCGAGTCGGCCCTCGAGGAGCCGGCCCCGGGCTGGATCGAGGGCGTCAAGGTCGCCGACGCCATCATCCTCGCCTACGCCCGCGAGAAGGTCACCCTCTTCCCGGGCAAGCGCTCGGGCATCATCGACGTCATCCCGGTCGACCTCGTCGCCAACTCGATCATCCTCTCGCTCGCCGAGGCCCTCGGCGAGCCGGGCCGCCGCCGCATCTACCAGTGCTGCTCGGGCGGCGGCAACCCGATCTCGCTCGGCGAGTTCATCGACCACCTCATGGCCGAGTCGAAGGCCAACTACGCCGCCTACGACCACCTCTTCTACCGCCAGCCGTCGAAGCCGTTCCTCGCCGTCAACCGCGCCCTCTTCGACCTCGTCATCTCGGGCGTCCGCCTCCCGCTCTCGCTCACCGACCGCGTCCTCAAGCTCCTCGGCAACTCGCGCGACCTCAAGATGCTCCGCAACCTCGACACCACCCAGTCGCTCGCCACCATCTTCGGCTTCTACACCGCCCCGGACTACATCTTCCGCAACGACGAGCTCATGGCCCTCGCCAACCGCATGGGCGAGGTCGACAAGGGCCTCTTCCCGGTCGACGCCCGCCTCATCGACTGGGAGCTCTACCTCCGCAAGATCCACCTCGCCGGCCTCAACCGCTACGCCCTCAAGGAGCGCAAGGTCTACTCGCTCAAGACCGCCCGCCAGCGCAAGAAGGCCGCCTAG |
| *SpCas9* | ATGCCCAAGAAGAAGCGCAAAGTCATGGACAAGAAGTACTCGATCGGCCTCGACATCGGCACCAACTCGGTCGGCTGGGCCGTCATCACCGACGAGTACAAGGTCCCGTCGAAGAAGTTCAAGGTCCTCGGCAACACCGACCGCCACTCGATCAAGAAGAACCTCATCGGCGCCCTCCTCTTCGACTCGGGCGAGACGGCGGAGGCCACCCGCCTCAAGCGCACGGCCCGCCGCCGATACACCCGCCGCAAGAACCGCATCTGCTACCTCCAGGAAATCTTCTCGAACGAGATGGCGAAGGTGGACGACTCGTTCTTCCACCGCCTCGAAGAGTCGTTCCTCGTCGAGGAAGACAAGAAGCACGAGCGCCACCCGATCTTCGGCAACATCGTGGACGAGGTCGCCTACCACGAGAAGTACCCCACCATCTACCACCTCCGCAAGAAGCTCGTGGACTCGACCTACAAGGCGGACCTCCGCCTCATCTACCTCGCCCTCGCGCACATGATCAAGTTCCGCGGCCACTTCCTCATCGAGGGCGACCTCAACCCGGACAACTCGGACGTGGACAAGCTCTTCATCCAGCTCGTCCAGACCTACAACCAGCTCTTCGAGGAGAACCCCATCAACGCCTCGGGCGTGGACGCCAAGGCCATCCTCTCGGCCCGCCTCTCGAAGTCGCGCCGCCTCGAAAACCTCATCGCCCAGCTCCCGGGCGAGAAGAAGAACGGCCTCTTCGGCAACCTCATCGCCCTCTCGCTCGGCCTCACCCCCAACTTCAAGTCGAACTTCGACCTCGCCGAGGACGCGAAGCTCCAGCTCTCGAAGGACACCTACGACGACGACCTCGACAACCTCCTCGCCCAGATCGGCGACCAGTACGCGGACCTCTTCCTCGCCGCGAAGAACCTCTCGGACGCCATCCTCCTCTCGGACATCCTCCGCGTCAACACCGAGATCACCAAGGCCCCGCTCTCGGCGTCGATGATCAAGCGCTACGACGAGCACCACCAGGACCTCACGCTCCTCAAGGCGCTCGTCCGCCAGCAGCTCCCCGAGAAGTACAAGGAAATCTTCTTCGACCAGTCGAAGAACGGCTACGCGGGCTACATCGACGGCGGCGCGTCGCAGGAAGAGTTCTACAAGTTCATCAAGCCGATCCTCGAAAAGATGGACGGCACCGAGGAACTCCTCGTCAAGCTCAACCGCGAGGACCTCCTCCGCAAGCAGCGCACCTTCGACAACGGCTCGATCACCCACCAGATCCACCTCGGCGAACTCCACGCCATCCTCCGCCGCCAGGAAGACTTCTACCCGTTCCTCAAGGACAACCGCGAGAAGATCGAGAAGATCCTCACCTTCCGCATCCCGTACTACGTCGGCCCCCTCGCCCGCGGCAACTCGCGCTTCGCGTGGATGACCCGCAAGTCGGAGGAGACCATCACCCCCTGGAACTTCGAGGAAGTCGTGGACAAGGGCGCCTCGGCCCAGTCGTTCATCGAGCGCATGACCAACTTCGACAAGAACCTCCCGAACGAGAAGGTCCTCCCCAAGCACTCGCTCCTCTACGAGTACTTCACCGTCTACAACGAACTCACCAAGGTCAAGTACGTCACCGAGGGCATGCGCAAGCCGGCCTTCCTCTCGGGCGAGCAGAAGAAGGCGATCGTGGACCTCCTCTTCAAGACCAACCGCAAGGTCACCGTCAAGCAGCTCAAGGAAGACTACTTCAAGAAGATCGAGTGCTTCGACTCGGTCGAGATCAGCGGCGTCGAGGACCGCTTCAACGCGTCGCTCGGCACCTACCACGACCTCCTCAAGATCATCAAGGACAAGGACTTCCTCGACAACGAGGAGAACGAGGACATCCTCGAAGACATCGTCCTCACCCTCACCCTCTTCGAGGACCGCGAGATGATCGAGGAGCGCCTCAAGACCTACGCCCACCTCTTCGACGACAAGGTCATGAAGCAGCTCAAGCGCCGCCGATACACGGGCTGGGGCCGCCTCTCGCGCAAGCTCATCAACGGCATCCGCGACAAGCAGTCGGGCAAGACCATCCTCGACTTCCTCAAGTCGGACGGCTTCGCCAACCGCAACTTCATGCAGCTCATCCACGACGACTCGCTCACCTTCAAGGAAGACATCCAGAAGGCCCAGGTCTCGGGCCAGGGCGACTCGCTCCACGAGCACATCGCCAACCTCGCCGGCTCGCCCGCGATCAAGAAGGGCATCCTCCAGACCGTCAAGGTCGTGGACGAACTCGTCAAGGTCATGGGCCGCCACAAGCCCGAGAACATCGTCATCGAGATGGCCCGCGAGAACCAGACCACCCAGAAGGGCCAGAAGAACTCGCGCGAGCGCATGAAGCGCATCGAGGAAGGCATCAAGGAACTCGGCTCGCAGATCCTCAAGGAGCACCCCGTCGAGAACACCCAGCTCCAGAACGAGAAGCTCTACCTCTACTACCTCCAGAACGGCCGCGACATGTACGTGGACCAGGAACTCGACATCAACCGCCTCTCGGACTACGACGTGGACCACATCGTCCCGCAGTCGTTCCTCAAGGACGACTCGATCGACAACAAGGTCCTCACCCGCTCGGACAAGAACCGCGGCAAGTCGGACAACGTCCCCTCGGAGGAAGTCGTCAAGAAGATGAAGAACTACTGGCGCCAGCTCCTCAACGCCAAGCTCATCACCCAGCGCAAGTTCGACAACCTCACGAAGGCGGAGCGCGGCGGACTCTCGGAACTCGACAAGGCGGGCTTCATCAAGCGCCAGCTCGTCGAGACCCGCCAGATCACCAAGCACGTCGCGCAGATCCTCGACTCGCGCATGAACACCAAGTACGACGAGAACGACAAGCTCATCCGCGAGGTCAAGGTCATCACCCTCAAGTCGAAGCTCGTCTCGGACTTCCGCAAGGACTTCCAGTTCTACAAGGTCCGCGAGATCAACAACTACCACCACGCCCACGACGCCTACCTCAACGCGGTCGTCGGCACCGCGCTCATCAAGAAGTACCCGAAGCTCGAATCGGAGTTCGTCTACGGCGACTACAAGGTCTACGACGTCCGCAAGATGATCGCCAAGTCGGAGCAGGAGATCGGCAAGGCCACCGCGAAGTACTTCTTCTACTCGAACATCATGAACTTCTTCAAGACCGAGATCACCCTCGCCAACGGCGAGATCCGCAAGCGCCCCCTCATCGAGACCAACGGCGAGACGGGCGAGATCGTCTGGGACAAGGGCCGCGACTTCGCCACCGTCCGCAAGGTCCTCTCGATGCCGCAGGTCAACATCGTCAAGAAGACCGAGGTCCAGACCGGCGGCTTCTCGAAGGAGTCGATCCTCCCCAAGCGCAACTCGGACAAGCTCATCGCCCGCAAGAAGGACTGGGACCCGAAGAAGTACGGCGGATTCGACTCGCCGACGGTCGCCTACTCGGTCCTCGTCGTCGCGAAGGTCGAGAAGGGCAAGTCGAAGAAGCTCAAGTCGGTCAAGGAACTCCTCGGCATCACCATCATGGAGCGCTCGTCGTTCGAGAAGAACCCGATCGACTTCCTCGAAGCGAAGGGCTACAAGGAAGTCAAGAAGGACCTCATCATCAAGCTCCCCAAGTACTCGCTCTTCGAGTTGGAGAACGGCCGCAAGCGCATGCTCGCCTCGGCCGGCGAACTCCAGAAGGGCAACGAACTCGCCCTCCCGTCGAAGTACGTCAACTTCCTCTACCTCGCGTCGCACTACGAGAAGCTCAAGGGCTCGCCCGAGGACAACGAGCAGAAGCAGCTCTTCGTCGAGCAGCACAAGCACTACCTCGACGAGATCATCGAGCAGATCAGCGAGTTCTCGAAGCGCGTCATCCTCGCCGACGCGAACCTCGACAAGGTCCTCTCGGCCTACAACAAGCACCGCGACAAGCCCATCCGCGAGCAGGCGGAGAACATCATCCACCTCTTCACCCTCACCAACCTCGGCGCCCCTGCCGCGTTCAAGTACTTCGACACCACCATCGACCGCAAGCGCTACACCTCGACCAAGGAAGTCCTCGACGCGACCCTCATCCACCAGTCGATCACCGGCCTCTACGAGACGCGCATCGACCTCTCGCAGCTCGGCGGCGACGAGATCCGCGAACTCAAGCGCCGCCTCGGCGAGTGCGAGGCGGGCGACGCGGGCACCCGCAAGCGCGTCAAGTACGAGTAG |
| *Yl G6PD1* | ATGACCGGCACCCTCCCGAAGTTCGGCGACGGCACCACCATCGTCGTCCTCGGCGCCTCGGGCGACCTCGCCAAGAAGAAGACCTTCCCGGCCCTCTTCGGCCTCTACCGCAACGGCCTCCTCCCGAAGAACGTCGAGATCATCGGCTACGCCCGCTCGAAGATGACCCAGGAGGAGTACCACGAGCGCATCTCGCACTACTTCAAGACCCCGGACGACCAGTCGAAGGAGCAGGCCAAGAAGTTCCTCGAGAACACCTGCTACGTCCAGGGCCCGTACGACGGCGCCGAGGGCTACCAGCGCCTCAACGAGAAGATCGAGGAGTTCGAGAAGAAGAAGCCGGAGCCGCACTACCGCCTCTTCTACCTCGCCCTCCCGCCGTCGGTCTTCCTCGAGGCCGCCAACGGCCTCAAGAAGTACGTCTACCCGGGCGAGGGCAAGGCCCGCATCATCATCGAGAAGCCGTTCGGCCACGACCTCGCCTCGTCGCGCGAGCTCCAGGACGGCCTCGCCCCGCTCTGGAAGGAGTCGGAGATCTTCCGCATCGACCACTACCTCGGCAAGGAGATGGTCAAGAACCTCAACATCCTCCGCTTCGGCAACCAGTTCCTCTCGGCCGTCTGGGACAAGAACACCATCTCGAACGTCCAGATCTCGTTCAAGGAGCCGTTCGGCACCGAGGGCCGCGGCGGCTACTTCAACGACATCGGCATCATCCGCGACGTCATCCAGAACCACCTCCTCCAGGTCCTCTCGATCCTCGCCATGGAGCGCCCGGTCACCTTCGGCGCCGAGGACATCCGCGACGAGAAGGTCAAGGTCCTCCGCTGCGTCGACATCCTCAACATCGACGACGTCATCCTCGGCCAGTACGGCCCGTCGGAGGACGGCAAGAAGCCGGGCTACACCGACGACGACGGCGTCCCGGACGACTCGCGCGCCGTCACCTTCGCCGCCCTCCACCTCCAGATCCACAACGACCGCTGGGAGGGCGTCCCGTTCATCCTCCGCGCCGGCAAGGCCCTCGACGAGGGCAAGGTCGAGATCCGCGTCCAGTTCCGCGACGTCACCAAGGGCGTCGTCGACCACCTCCCGCGCAACGAGCTCGTCATCCGCATCCAGCCGTCGGAGTCGATCTACATGAAGATGAACTCGAAGCTCCCGGGCCTCACCGCCAAGAACATCGTCACCGACCTCGACCTCACCTACAACCGCCGCTACTCGGACGTCCGCATCCCGGAGGCCTACGAGTCGCTCATCCTCGACTGCCTCAAGGGCGACCACACCAACTTCGTCCGCAACGACGAGCTCGACATCTCGTGGAAGATCTTCACCGACCTCCTCCACAAGATCGACGAGGACAAGTCGATCGTCCCGGAGAAGTACGCCTACGGCTCGCGCGGCCCGGAGCGCCTCAAGCAGTGGCTCCGCGACCGCGGCTACGTCCGCAACGGCACCGAGCTCTACCAGTGGCCGGTCACCAAGGGCTCGTCGTAG |
| *Yl ME1* | ATGCTCCGCCTCCGCACCATGCGCCCGACCCAGACCTCGGTCCGCGCCGCCCTCGGCCCGACCGCCGCCGCCCGCAACATGTCGTCGTCGTCGCCGTCGTCGTTCGAGTACTCGTCGTACGTCAAGGGCACCCGCGAGATCGGCCACCGCAAGGCCCCGACCACCCGCCTCTCGGTCGAGGGCCCGATCTACGTCGGCTTCGACGGCATCCGCCTCCTCAACCTCCCGCACCTCAACAAGGGCTCGGGCTTCCCGCTCAACGAGCGCCGCGAGTTCCGCCTCTCGGGCCTCCTCCCGTCGGCCGAGGCCACCCTCGAGGAGCAGGTCGACCGCGCCTACCAGCAGTTCAAGAAGTGCGGCACCCCGCTCGCCAAGAACGGCTTCTGCACCTCGCTCAAGTTCCAGAACGAGGTCCTCTACTACGCCCTCCTCCTCAAGCACGTCAAGGAGGTCTTCCCGATCATCTACACCCCGACCCAGGGCGAGGCCATCGAGCAGTACTCGCGCCTCTTCCGCCGCCCGGAGGGCTGCTTCCTCGACATCACCTCGCCGTACGACGTCGAGGAGCGCCTCGGCGCCTTCGGCGACCACGACGACATCGACTACATCGTCGTCACCGACTCGGAGGGCATCCTCGGCATCGGCGACCAGGGCGTCGGCGGCATCGGCATCTCGATCGCCAAGCTCGCCCTCATGACCCTCTGCGCCGGCGTCAACCCGTCGCGCGTCATCCCGGTCGTCCTCGACACCGGCACCAACAACCAGGAGCTCCTCCACGACCCGCTCTACCTCGGCCGCCGCATGCCGCGCGTCCGCGGCAAGCAGTACGACGACTTCATCGACAACTTCGTCCAGTCGGCCCGCCGCCTCTACCCGAAGGCCGTCATCCACTTCGAGGACTTCGGCCTCGCCAACGCCCACAAGATCCTCGACAAGTACCGCCCGGAGATCCCGTGCTTCAACGACGACATCCAGGGCACCGGCGCCGTCACCCTCGCCTCGATCACCGCCGCCCTCAAGGTCCTCGGCAAGAACATCACCGACACCCGCATCCTCGTCTACGGCGCCGGCTCGGCCGGCATGGGCATCGCCGAGCAGGTCTACGACAACCTCGTCGCCCAGGGCCTCGACGACAAGACCGCCCGCCAGAACATCTTCCTCATGGACCGCCCGGGCCTCCTCACCACCGCCCTCACCGACGAGCAGATGTCGGACGTCCAGAAGCCGTTCGCCAAGGACAAGGCCAACTACGAGGGCGTCGACACCAAGACCCTCGAGCACGTCGTCGCCGCCGTCAAGCCGCACATCCTCATCGGCTGCTCGACCCAGCCGGGCGCCTTCAACGAGAAGGTCGTCAAGGAGATGCTCAAGCACACCCCGCGCCCGATCATCCTCCCGCTCTCGAACCCGACCCGCCTCCACGAGGCCGTCCCGGCCGACCTCTACAAGTGGACCGACGGCAAGGCCCTCGTCGCCACCGGCTCGCCGTTCGACCCGGTCAACGGCAAGGAGACCTCGGAGAACAACAACTGCTTCGTCTTCCCGGGCATCGGCCTCGGCGCCATCCTCTCGCGCTCGAAGCTCATCACCAACACCATGATCGCCGCCGCCATCGAGTGCCTCGCCGAGCAGGCCCCGATCCTCAAGAACCACGACGAGGGCGTCCTCCCGGACGTCGCCCTCATCCAGATCATCTCGGCCCGCGTCGCCACCGCCGTCGTCCTCCAGGCCAAGGCCGAGGGCCTCGCCACCGTCGAGGAGGAGCTCAAGCCGGGCACCAAGGAGCACGTCCAGATCCCGGACAACTTCGACGAGTGCCTCGCCTGGGTCGAGACCCAGATGTGGCGCCCGGTCTACCGCCCGCTCATCCACGTCCGCGACTACGACTAG |

**Table S2.** List of gRNAs used in this study.

| **gRNA** | **Target sequence** | **Knockout rate** | **Mutation** |
| --- | --- | --- | --- |
| **CAR2** | GTGCTCCAGAGGACTAGCGC | 195/197 | 19 bp deletion |
| **LRO1-1** | GAGTTCGGTGACGTTGAGAG | 0/7 | - |
| **LRO1-2** | GGCGAGATGCGGCTGCGCGA | 3/7 | 16 bp deletion |
| **DGA1-1** | TTGCGCCCTTTGGCGTCCCG | 2/7 | 13 bp deletion |
| **ARE1-1** | TGCCGAGTCGGGGTGTCGGA | 4/7 | 10 bp deletion |
| **13524-1** | GGAGATTCCTGCGTAGCGCG | 1/7 | 5 bp deletion |
| **11642-1** | CATCGGTCGTGTTTGCGCGG | 2/7 | 11 bp deletion |
| **11216-1** | CGAGTTGAGCGTGCTCGCGA | 3/7 | 1 bp deletion |
| **16017-1** | CGCCGCTCGCGCAAGAGCCG | 0/14 | - |
| **9637-1** | GAGGAAGGTCGTGCAAGCGG | 0/14 | - |
| **9637-2** | TCTGCGAGCGAAAGGCGGAG | 7/7 | 1 bp deletion |
| **13167-1** | AGATGTGGCTGAGAAGAGTG | 2/7 | 1 bp deletion |

**Table S3.** List of Mycocosm proteinIDs for *R. toruloides* IFO0880 gene targets in this study.

| **Gene** | **ProteinID** |
| --- | --- |
| *ACC1* | 8639 |
| *ACL1* | 9726 |
| *G6PD1* | 10000 |
| *FAA1* | 11167 |
| *TGL2* | 14617 |
| *AMPD1* | 11469 |
| *SCD1* | 9730 |
| *DGA1* | 16460 |
| *LRO1* | 16477 |
| *ARE1* | 11799 |
| *FAO* | 11216 |
| *FAO* | 11642 |
| *FAO* | 13524 |
| *PXA1* | 9637 |
| *PXA1* | 13167 |

**Table S4**. List of *R. toruloides* strains used in this study. NTC, nourseothricin; HYG, hygromycin.

| **Strain Name and Genotype** | **Parent strain** | **Antibiotic** | **Source** |
| --- | --- | --- | --- |
| IFO0880 | N/A | N/A | [1] |
| IFO0880-pTEF1-MaFAR | IFO0880 | NTC | This study |
| IFO0880-pGAPDH1-MaFAR | IFO0880 | NTC | This study |
| IFO0880-pGAPDH1-SpCas9 | IFO0880 | G418 | This study |
| IFO0880-p27-SpCas9 | IFO0880 | G418 | This study |
| IFO0880-p17-SpCas9 | IFO0880 | G418 | This study |
| IFO0880-pANT1-SpCas9 | IFO0880 | G418 | This study |
| IFO0880-pTEF1-SpCas9 | IFO0880 | G418 | This study |
| 880CF (IFO0880-pANT1-SpCas9-pGAPDH1-MaFAR) | IFO0880 | G418 | This study |
| 880CF-p17-G6PD1 | 880CF | G418, NTC | This study |
| 880CF-p17-ACL1 | 880CF | G418, NTC | This study |
| 880CF-p17-SCD1 | 880CF | G418, NTC | This study |
| 880CF-p17-AMPD1 | 880CF | G418, NTC | This study |
| 880CF-p17-YlG6PD1 | 880CF | G418, NTC | This study |
| 880CF-p17-YlME1 | 880CF | G418, NTC | This study |
| 880CF-pGAPDH1-ACC1 | 880CF | G418, NTC | This study |
| 880CF- pANT1-FAA1-pTEF1-TGL2 | 880CF | G418, NTC | This study |
| 880CF-LRO1Δ | 880CF | G418, HYG | This study |
| 880CF-DGA1Δ | 880CF | G418, HYG | This study |
| 880CF-ARE1Δ | 880CF | G418, HYG | This study |
| 880CF-FAO/11216Δ | 880CF | G418, HYG | This study |
| 880CF-FAO/11642Δ | 880CF | G418, HYG | This study |
| 880CF-FAO/13524Δ | 880CF | G418, HYG | This study |
| 880CF-PXA1/9637Δ | 880CF | G418, HYG | This study |
| 880CF-PXA1/13167Δ | 880CF | G418, HYG | This study |
| 880CF-LRO1Δ-p17-ACL1 | 880CF-LRO1Δ | G418, HYG, NTC | This study |
| 880CF-LRO1Δ-pGAPDH1-ACC1 | 880CF-LRO1Δ | G418, HYG, NTC | This study |
| 880CF-LRO1Δ-pGAPDH1-ACC1-p17-ACL1 | 880CF-LRO1Δ | G418, HYG, NTC | This study |
| 880CF-DGA1Δ-p17-ACL1 | 880CF-DGA1Δ | G418, HYG, NTC | This study |
| 880CF-DGA1Δ-pGAPDH1-ACC1 | 880CF-DGA1Δ | G418, HYG, NTC | This study |
| 880CF-DGA1Δ-pGAPDH1-ACC1-p17-ACL1 | 880CF-DGA1Δ | G418, HYG, NTC | This study |

**Table S5.** Ion abundances, as measured by direct infusion mass spectrometry, of lipid species in 880CF, 880CF-*LRO1*Δ, and 880CF-*DGA1*Δ following 72-hours culture in pH 7-adjusted SC media with 4% glucose, following inoculation to an initial OD of 0.2. Error bars represent standard deviation of biological triplicates. Cer, Ceramide; PE, phosphatidylethanolamine; PC, phosphatidylcholine; LPC, lyso-phosphatidylcholine; DAG, diacylglyceride; TAG, triacylglyceride; LPE, lyso-phosphatidylethanolamine; MIPC, mannosylinositol phosphorylceramide; PI, phosphatidylinositol; CL, cardiolipin; IPC, inositolphosphoryl-ceramide; PA, phosphatidic acid; PS, phosphatidylserine; MIP2C, mannosyl-diphosphoinositol-ceramide; PG, phosphatidylglycerol.

|  | **880CF** | **880CF-DGA1∆** | **880CF-LRO1∆** |
| --- | --- | --- | --- |
| **Cer** | 8.0±0.82 | 18.2±7.2 | 7.1±1.1 |
| **PE** | 701.1±24.7 | 1208.7±41.1 | 878.5±34.9 |
| **PC** | 741.1±22.6 | 753.1±19.5 | 879.0±17.5 |
| **LPC** | 222.5±14.9 | 280.9±78.6 | 210.7±40.6 |
| **EE** | 198.7±27.9 | 125.4±18.2 | 151.3±15.6 |
| **DAG** | 437.2±15.9 | 355.3±13.9 | 750.8±16.9 |
| **TAG** | 10358.5±1160.0 | 2008.0±33.9 | 16682.4±602.5 |
| **LPE** | 124.0±28.0 | 122.1±12.6 | 91.3±16.0 |
| **MIPC** | 8.4±0.67 | 6.9±1.9 | 5.2±1.2 |
| **PI** | 261.5±30.2 | 298.6±11.0 | 203.8±6.1 |
| **CL** | 1048.0±155.8 | 818.3±109.5 | 436.3±58.1 |
| **IPC** | 16.4±3.3 | 10.2±1.7 | 12.5±2.0 |
| **PA** | 22.9±0.99 | 27.1±0.97 | 20.5±0.96 |
| **PS** | 2.3±0.3 | 2.6±0.2 | 11.6±17.2 |
| **MIP2C** | 33.1±11.0 | 54.6±14.1 | 37.4±7.5 |
| **PG** | 8.7±0.94 | 13.0±0.83 | 5.9±0.29 |

**Table S6.** List of primers used in this study.

| **Primer** | **Sequence** | **Notes** |
| --- | --- | --- |
| ZPK F | CAAATTGACGCTTAGACAAC | Gene expression cassette amplification for transformation |
| ZPK R | TATATCCTGTCAAACACTGATAG |  |
| gRNA F | GACTATTTGCAAAGGGAAGGG | gRNA expression cassette amplification for transformation |
| gRNA R | TTTTTGTGATGCTCGTCAG |  |
| pANT1 F | CAACAGGATTCAATCTTAAGCAAGTCAAGATTCTGCGG | pANT1 cloning |
| pANT1 R | TTCTTCTTGGGCATCAATTGGGCTGAACAAAGTTTTCC |  |
| p17 F | CAACAGGATTCAATCTTAAGGGGGGACAGTGCATG | p17 cloning |
| p17 R | TTCTTCTTGGGCATCAATTGGTTGAGTGACGAGGA |  |
| pTEF1 F | CAACAGGATTCAATCTTAAGCGCGAAGCGGTAGA | pTEF1 cloning |
| pTEF1 R | TTCTTCTTGGGCATCAATTGCTATCCCCCCCAGTA |  |
| G6PD F | AAACAATTGATGAAGAAGAAGCTCGAAGAGTTCCTC | G6PD1 cloning |
| G6PD R | AAAACTAGTGCAAGAACAAGTTCGTTTTAATTGCTGCAC |  |
| ACL1 F | AAACAATTGATGTCGGCGAAGGTGCG | ACL1 cloning |
| ACL1 R | AAAACTAGTTTACTGGCGCTGCTGGAC |  |
| FAA1 F | ACTCAACCAATTGATGCCTGGACGATACGCTTC | FAA1 cloning |
| FAA1 R | TGTTTGAACGATCTCACGGGTATACCTTCTGC |  |
| TGL2 F | CTGGGGGGGATAGATGGGCGACAACTACAAG | TGL2 cloning |
| TGL2 R | GGAGAAAACTAGTTCAAACTCGGTCGTGCTTG |  |
| SCD1 F | AAACAATTGATGACTGCCTCGTCGGC | SCD1 cloning |
| SCD1 R | AAAACTAGTTTACGCCTTGACCGTCAGGC |  |
| AMPD1 F | AAACAATTGATGGCTGACACAATAGATTCACTGCGA | AMPD1 cloning |
| AMPD1 R | AAAACTAGTCTACCTCCCATTCACGCTGG |  |
| sgLRO1 F1 | GGGAGAGTTCGGTGACGTTGAGAG | LRO1 gRNA cloning |
| sgLRO1 R1 | AAACCTCTCAACGTCACCGAACTC |  |
| sgLRO1 F2 | GGGAGGCGAGATGCGGCTGCGCGA | LRO1 gRNA cloning |
| sgLRO1 R2 | AAACTCGCGCAGCCGCATCTCGCC |  |
| sgDGA1 F1 | GGGATTGCGCCCTTTGGCGTCCCG | DGA1 gRNA cloning |
| sgDGA1 R1 | AAACCGGGACGCCAAAGGGCGCAA |  |
| SgDGA F2 | GGGAGTCTTCGGCTATCATCCGCA | DGA1 gRNA cloning |
| SgDGA R2 | AAACTGCGGATGATAGCCGAAGAC |  |
| SgARE1 F1 | GGGATGCCGAGTCGGGGTGTCGGA | ARE1 gRNA cloning |
| SgARE1 R1 | AAACTCCGACACCCCGACTCGGCA |  |
| SgARE1 F2 | GGGATGTCCGCGCGAAGTTGCGGA | ARE1 gRNA cloning |
| SgARE1 R2 | AAACTCCGCAACTTCGCGCGGACA |  |
| SgFAO 13524 F1 | GGGAGGAGATTCCTGCGTAGCGCG | FAO gRNA cloning |
| SgFAO 13524 R1 | AAACCGCGCTACGCAGGAATCTCC |  |
| SgFAO 11642 F1 | GGGACATCGGTCGTGTTTGCGCGG | FAO gRNA cloning |
| SgFAO 11642 R1 | AAACCCGCGCAAACACGACCGATG |  |
| SgFAO 11216 F1 | GGGACGAGTTGAGCGTGCTCGCGA | FAO gRNA cloning |
| SgFAO 11216 R1 | AAACTCGCGAGCACGCTCAACTCG |  |
| SgPEX10 16017 F1 | GGGACGCCGCTCGCGCAAGAGCCG | PEX10 gRNA cloning |
| SgPEX10 16017 R1 | AAACCGGCTCTTGCGCGAGCGGCG |  |
| SgPXA1 9637 F1 | GGGAGAGGAAGGTCGTGCAAGCGG | PXA1 gRNA cloning |
| SgPXA1 9637 R1 | AAACCCGCTTGCACGACCTTCCTC |  |
| SgPXA1 13167 F1 | GGGAAGATGTGGCTGAGAAGAGTG | PXA1 gRNA cloning |
| SgPXA1 13167 R1 | AAACCACTCTTCTCAGCCACATCT |  |
| SgPXA 9637 F2 | GGGATCTGCGAGCGAAAGGCGGAG | PXA1 gRNA cloning |
| SgPXA 9637 R2 | AAACCTCCGCCTTTCGCTCGCAGA |  |
| LRO1 Seq F | CTTAAAATGCGTCCAGACTGA | LRO1 gDNA sequencing |
| LRO1 Seq R | CCTTGACCCAAGCCTCC |  |
| DGA seq F | TTCGCCTCGTCCTCCCGC | DGA1 gDNA sequencing |
| DGA seq R | AGGATGTTCTGGCAGCTCTTCATCG |  |
| ARE1 Seq F | GACGCTCTTTCTCCTCCT | ARE1 gDNA sequencing |
| ARE1 Seq R | GAGCTGTGAGTCCGAAG |  |
| PXA1 13167 Seq F | ACTACATCCAGCACAGGAGGAGGTAC | PXA1 gDNA sequencing |
| PXA1 13137 Seq R | TCCGCCAATGACGTGCTAAACTTCTG |  |
| PXA1 9637 Seq F | ATGGCACCCGCACAGTCC | PXA1 gDNA sequencing |
| PXA1 9637 Seq R | ATTCTCGAATCAAGATTGACGACCTTATAGAACGTCG |  |
| PEX10 Seq F | ATGCCAGACTCGCCAGCG | PEX10 gDNA sequencing |
| PEX10 Seq R | ACGAGACGAAGGACGGAGCAAC |  |
| FAO 11216 Seq F | ATGGCTCCCCGACAGCG | FAO gDNA sequencing |
| FAO 11216 Seq R | GGGAGAGGAGAAGACGCACGG |  |
| FAO 11642 Seq F | ATGGTCGCACCAACAACGCTG | FAO gDNA sequencing |
| FAO 11642 Seq R | TCCCGTAAGGACGACGAGGTTCTG |  |
| FAO 13524 Seq F | ATGCCCCTCTCGCTCCCA | FAO gDNA sequencing |
| FAO 13524 Seq R | ACGAGAGAAGAGGAGCTTTTGGTCGG |  |

**Table S7.** List of plasmids used in this study.

| **Plasmid name** | **Backbone** | **Yeast selection marker** | **Expression cassette** | **Source** |
| --- | --- | --- | --- | --- |
| pRTN-pTEF1-MaFAR | pRTN | Nourseothricin | pTEF1-MaFAR-T35S | This study |
| pRTN-pGAPDH1-MaFAR | pRTN | Nourseothricin | pGAPDH1-MaFAR-T35S | This study |
| NM9-pGAPDH1-SpCas9 | NM9 | G418 | pGAPDH1-SpCas9-T35S | This study |
| NM9-p27-SpCas9 | NM9 | G418 | p27-SpCas9-T35S | This study |
| NM9-p17-SpCas9 | NM9 | G418 | p17-SpCas9-T35S | This study |
| NM9-pANT1-SpCas9 | NM9 | G418 | pANT1-SpCas9-T35S | This study |
| NM9-pTEF1-SpCas9 | NM9 | G418 | pTEF1-SpCas9-T35S | This study |
| NM9-pANT1-SpCas9-pGAPDH-MaFAR | NM9 | G418 | pANT1-SpCas9-T35S-pGAPDH-MaFAR-Ttub | This study |
| pRTN-p17-G6PD1 | pRTN | Nourseothricin | p17-G6PD1-T35S | This study |
| pRTN-p17-ACL1 | pRTN | Nourseothricin | p17-ACL1-T35S | This study |
| pRTN-p17-SCD1 | pRTN | Nourseothricin | p17-SCD1-T35S | This study |
| pRTN-p17-AMPD1 | pRTN | Nourseothricin | p17-AMPD1-T35S | This study |
| pRTN-p17-YlG6PD1 | pRTN | Nourseothricin | p17-YlG6PD1-T35S | This study |
| pRTN-p17-YlME1 | pRTN | Nourseothricin | p17-YlME1-T35S | This study |
| pRTN-pANT1-FAA1-Tnos-pTEF1-TGL2-T35S | pRTN | Nourseothricin | pANT1-FAA1-Tnos-pTEF1-TGL2-T35S | This study |
| pGI2_880_ACC | pGI2 | Nourseothricin | pGAPDH1-ACC1 | [1] |
| pRTH | pRTH | Hygromycin | 5S-tRNAtyr'-*Bsa*I-*Bsa*I-polyT | This study |
| pRTH-sgCAR2 | pRTH | Hygromycin | 5S-tRNAtyr'-sgCAR2-polyT | This study |
| pRTH-sgLRO1-1 | pRTH | Hygromycin | 5S-tRNAtyr'-sgLRO1-1-polyT | This study |
| pRTH-sgLRO1-2 | pRTH | Hygromycin | 5S-tRNAtyr'-sgLRO1-2-polyT | This study |
| pRTH-sgDGA1-1 | pRTH | Hygromycin | 5S-tRNAtyr'-sgDGA1-1-polyT | This study |
| pRTH-sgDGA1-2 | pRTH | Hygromycin | 5S-tRNAtyr'-sgDGA1-2-polyT | This study |
| pRTH-sgARE1-1 | pRTH | Hygromycin | 5S-tRNAtyr'-sgARE1-1-polyT | This study |
| pRTH-sgARE1-2 | pRTH | Hygromycin | 5S-tRNAtyr'-sgARE1-2-polyT | This study |
| pRTH-sgFAO-13524 | pRTH | Hygromycin | 5S-tRNAtyr'-sgFAO-13524-polyT | This study |
| pRTH-sgFAO-11642 | pRTH | Hygromycin | 5S-tRNAtyr'-sgFAO-11642-polyT | This study |
| pRTH-sgFAO-11216 | pRTH | Hygromycin | 5S-tRNAtyr'-sgFAO-11216-polyT | This study |
| pRTH-sgPEX10-16017 | pRTH | Hygromycin | 5S-tRNAtyr'-sgPEX10-16017-polyT | This study |
| pRTH-sgPXA1-9637-1 | pRTH | Hygromycin | 5S-tRNAtyr'-sgPXA1-9637-1-polyT | This study |
| pRTH-sgPXA1-9637-2 | pRTH | Hygromycin | 5S-tRNAtyr'-sgPXA1-9637-2-polyT | This study |
| pRTH-sgPXA1-13167 | pRTH | Hygromycin | 5S-tRNAtyr'-sgPXA1-13167-polyT | This study |

**Table 87.** List of lipidomics internal standards and their absolute amounts used for spike-in. Cer, Ceramide; PE, phosphatidylethanolamine; PC, phosphatidylcholine; LPC, lyso-phosphatidylcholine; DAG, diacylglyceride; TAG, triacylglyceride; LPE, lyso-phosphatidylethanolamine; MIPC, mannosylinositol phosphorylceramide; PI, phosphatidylinositol; CL, cardiolipin; IPC, inositolphosphoryl-ceramide; PA, phosphatidic acid; PS, phosphatidylserine; MIP2C, mannosyl-diphosphoinositol-ceramide; PG, phosphatidylglycerol.

| Class | Component | Amount (pmol) | Mode |
| --- | --- | --- | --- |
| PC | IS PC 15:0/18:1-d7 | 200.1 | Pos |
| PE | IS PE 17:0/14:1 | 14.58 | Pos |
| PS | IS PS 17:0/14:1 | 13.44 | Neg |
| PG | IS PG 15:0/18:1-d7 | 34.96 | Neg |
| PI | IS PI 17:0/14:1 | 12.59 | Neg |
| PA | IS PA 15:0/18:1-d7 | 10 | Neg |
| LPC | IS LPC 18:1-d7 | 45 | Pos |
| LPE | IS LPE 18:1-d7 | 10.075 | Pos |
| EE | IS CE 18:1-d7 | 500.4 | Pos |
| DAG | IS DAG 15:0/18:1-d7 | 14.977 | Pos |
| TAG | IS TAG 15:0/18:1-d7/15:0 | 65.043 | Pos |
| Cer | IS Cer 18:1;2/17:0 | 45.295 | Pos |
| Ergosterol | IS Cholesterol-d7 | 250.12 | Pos |

**References**

1. Zhang S, Skerker JM, Rutter CD, Maurer MJ, Arkin AP, Rao C V. Engineering *Rhodosporidium toruloides* for increased lipid production. *Biotechnol Bioeng*. 113:1056–66 (2016).
